# Thermodynamics of Acidity and Photoacidity in *trans*-Resveratrol Analogues: The Role of Prototropic Tautomerism

**DOI:** 10.1021/acsomega.6c06306

**Published:** 2026-08-08

**Authors:** Andrea Kováčová, Martin Michalík, Vladimír Lukeš

**Affiliations:** Faculty of Chemical and Food Technology, Slovak University of Technology in Bratislava, Radlinského 9, Bratislava SK-812 37, Slovakia

## Abstract

The thermodynamics of protonation equilibria and equilibria between enol and keto tautomers occurring in naturally found hydroxy derivatives of *trans-*resveratrol analogues were investigated. Reaction Gibbs free energies at room temperature were obtained using the B3LYP functional together with a continuum solvation model for water. From these data, acidity constants (p*K*_a_) and tautomerization constants (p*K*_T_) were calculated for the electronic ground state (S_0_) and the lowest excited singlet state (S_1_). The results reveal a pronounced dependence of both equilibria on the position of the hydroxy substituents. Upon photoexcitation, the compounds become considerably stronger acids and indicate, in many cases, a shift toward the keto tautomers rather than the parent enol forms. Moreover, our results support the feasibility of proton transfer from a hydroxy group on one aromatic ring to the adjacent ring via the central −CH=CH– bridge. Such processes may be relevant for nonradiative de-excitation pathways and for the protective biochemical functions of these compounds in plants.

## Graphical abstract

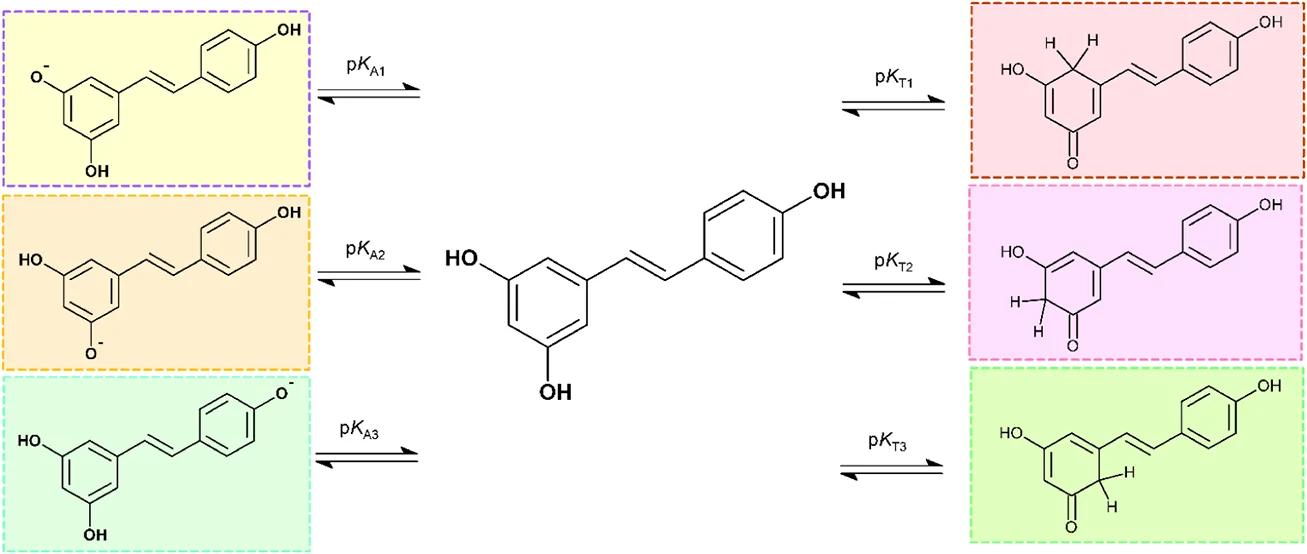

## 1 Introduction

Stilbenols are a class of organic molecules with remarkable biological and physicochemical properties. They belong to the broader family of stilbenes, characterized by a 1,2-diphenylethylene backbone (see Figure 1). Stilbenols represent hydroxylated derivatives of stilbene, bearing one or more phenolic hydroxy groups on aromatic rings. From a structural point of view, the 1,2-diphenylethylene (stilbene) scaffold consists of two benzene rings joined by an ethylene segment. There are two isomeric forms: (*E*)-stilbene (*trans*-stilbene), which is not sterically hindered, and (*Z*)-stilbene (*cis*-stilbene), which is less stable because of the steric interactions between the aromatic rings.^1^ The *E* form exhibits more potent anticancer activity and is thermodynamically more stable. The *E*- and *Z*-conformations exhibit different pharmacological activities. In natural systems, many of these are synthesized by plants, especially in rhubarb, grapes, berries, and peanuts. These compounds play an important role in plants, particularly in their defense mechanisms against reactive oxygen species or abiotic stress, such as drought, temperature extremes, and mechanical damage.^2,3^ Photoacidity and tautomerism constants are usually calculated from experimental spectra and their maximal peaks by Förster equations. Since significant uncertainty exists when peaks are estimated, this is also projected into less reliable calculated values. Theoretical thermodynamic calculations at the DFT level do not suffer from these shortcomings and might help with better qualitative trend estimation.

**Figure 1:**
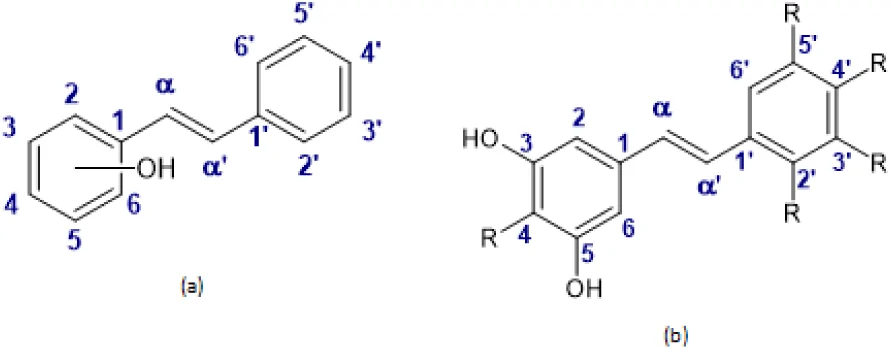
Carbon atom numbering and notation of studied stilbenols with (a) one, (b) two, and more OH groups on site R.

Like the parental stilbene, the *trans*-*cis* isomerization of stilbenols can occur upon optical excitations. In addition, photon absorption may be accompanied by photoacidity.^4^ Hydroxystilbenes, in their electronic ground state, are weak acids. For example, the negative logarithms of the experimental acid dissociation constant (p*K*_a_) in water for *trans*-3-hydroxystilbene (**3**) and *trans*-4-hydroxystilbene (**4**)^5^ equal to 10.1 and 9.3, respectively. Optical excitations of these molecules affect the acid–base equilibrium.^6^ Corresponding approximated p*K*_a_* values, determined from the Förster equation^7^ are 0.1 for compound **3** and 2.0 for compound **4**, respectively. The kinetic measurements performed on 3-hydroxystilbene showed an even higher p*K*_a_*, which was found to be 2.3.^5^ In the case of molecule **4**, the acidity constant could not be accurately determined. A possible explanation for these experimental errors is attributed to a difference in the torsional barriers for −C=C– torsion between the *trans* and *cis* conformations. Para substitution decreases the barrier and shortens the singlet lifetime. The excited-state proton-transfer (ESPT) process of stilbenes with more hydroxy groups can have greater practical importance. For example, *trans*-resveratrol (**35_4**) is known to transfer a proton to the acid-sensing chemoreceptor of the fungus, and this proton transfer accelerates the plant’s resistance to fungal infection.^8,9^

The presence of hydroxy group(s) on the aromatic nucleus provides conditions for prototropic tautomerism. This process is reversible, and it can proceed intra- or intermolecularly. The labile proton(s) can move from proton-donor site(s) to proton-acceptor site(s), leading to the generation of a mixture of two or more constitutional isomers, called tautomers (see Figure 3). The prototropic tautomerism^10^ of phenols exhibits a specific position among keto–enol equilibria occurring in organic compounds. Phenols, under standard conditions, prefer the enol form.^11^ When considering 2,4-cyclohexadienone or 2,5-cyclohexadienone,^12^ the corresponding enol tautomer is the thermodynamically more stable molecule with effective ***π***-electron delocalization. The appearance of phenol-dienone tautomerism can be modulated by the addition of another aromatic ring or substitution. The keto tautomers of phenols are considered as reactive intermediates in various reactions.^13–15^

**Figure 2:**
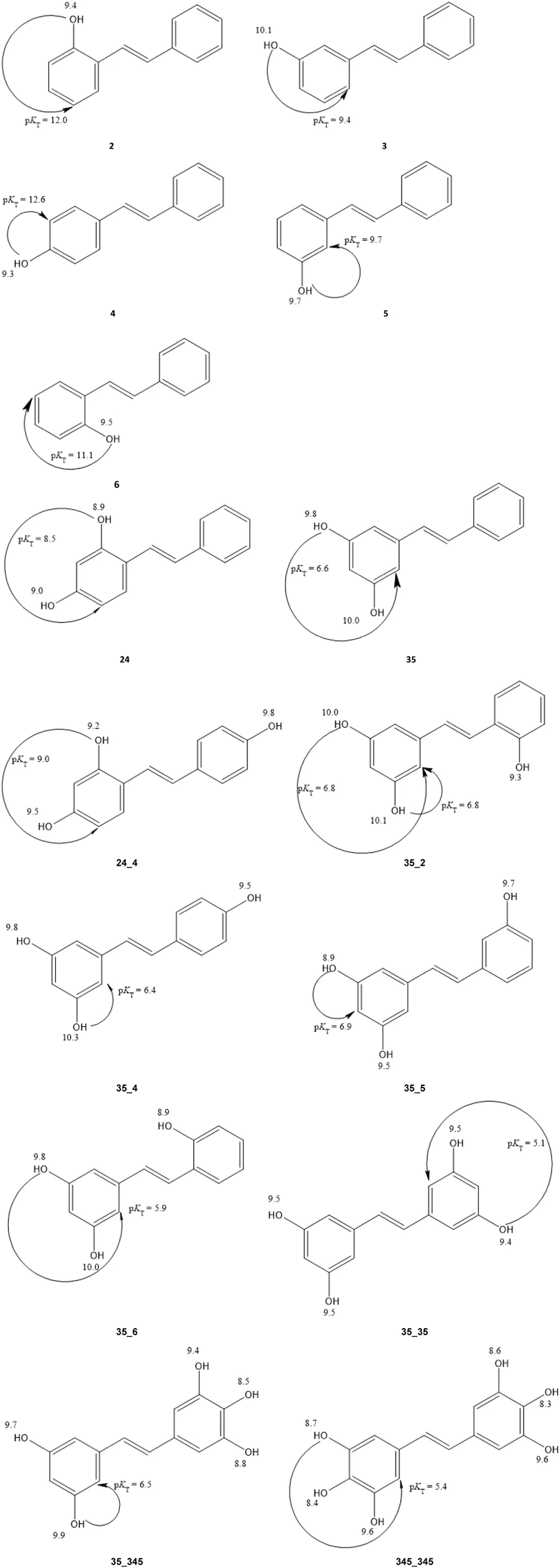
Scaled p*K*_a,sc_ values and the scaled p*K*_T,sc_ values for energetically preferred keto–enol tautomerization equilibria.

Although a similar process can be expected for hydroxystilbenes, the protonation equilibria and prototropic tautomerism have not yet been systematically studied, even at the quantum chemical theoretical level. Compared to substituted hydroxybenzenes, the question arises whether, from an energetic point of view, conditions may arise for the transfer of a proton from the hydroxy group to the distant aromatic ring of stilbene along the −CH=CH– bridge. With respect to such published facts and remaining open problems, we decided to study the thermodynamics of these processes for a selected group of naturally occurring *trans*-stilbenols (Figure 1). The results will be compared for the electronic ground (S_0_) and lowest energy (S_1_) states. The theoretical data obtained in this work will be contrasted with available published experimental results. For reference purposes, we will also perform calculations for phenol, 1-naphthol, and 2-naphthol. Experimental data for acid–base or tautomeric equilibrium in water have been published for these molecules.

## 2 Calculation Details

Although *trans*-stilbene is a relatively rigid planar system, the presence of hydroxy groups allows for the possibility of multiple conformations because of their internal rotation. In principle, two adjacent OH groups will form a stable intramolecular hydrogen bond. For this reason, we decided to perform conformational sampling at the extended tight-binding semiempirical GFN-xTB level of theory using the CREST program.^16^ The energetically preferred CREST conformers were then used as input geometries for optimization at the density functional theory (DFT) level. The energetically preferred conformation of the parent molecule was used as the starting geometry for the optimization of the rest of the molecules in the electronic S_0_ and S_1_ states. The DFT calculations were performed using the 3-parameter hybrid B3LYP functional^17^ combined with the 6–311++G** basis set^18,19^ of atomic orbitals. An implicit SMD (solvation model based on the quantum mechanical charge density of a solute molecule interacting with a continuum) approach^20^ was used to describe the solvent effect of water in all types of calculations. The excited-state geometries, as well as their corresponding de-excitation (emission) energies, were computed by the time-dependent (TD-)­DFT method.^21^ Optimum structures were confirmed to be real minima by vibrational analysis. The parent conformation that was thermodynamically most favorable was selected for further calculations. The Gibbs free energy was used as the energy criterion. All quantum chemical calculations were performed with the default settings of the Gaussian 16 program package, revision B.01.^22^ The selected combination of DFT functional and basis set represents an optimal selection between the computational requirements, numerical stability of calculations, and chemical precision with respect to experiments. Satisfactory results were obtained in calculations of thermodynamic quantities as well as electronic structure.^23–25^

The Gibbs free energies were computed for a temperature of 298.15 K and a pressure of 101.325 kPa. The value of −1121 kJ mol^–1^ was used for the Gibbs free energy of the hydrated proton^26^ representing the sum of the Gibbs free energy in a vacuum, *G*(H^+^) (−1115 kJ mol^–1^)^27^ and the corresponding solvation correction (−6 kJ mol^–1^).^28^ Since p*K*_a_ is defined for 1 mol L^–1^, the Gibbs free energies must be corrected according to the equation

1 $${G^{{}^{\circ}}}_{1M}={G^{{}^{\circ}}}_{1\mathrm{atm}}+\mathrm{RT}\;\ln\left(24.26\right)$$

For a temperature of 298.15 K, the correction is 7.9 kJ mol^–1^.^29^ The reaction dissociation Gibbs free energy (Δ_r_ *G*) was calculated as follows

2 $$\Delta_{r}G={G^{{}^{\circ}}}_{1M}\left(\mathrm{anion}\right)-{G^{{}^{\circ}}}_{1M}\left(\mathrm{enol}\right)+{G^{{}^{\circ}}}_{1M}\left(H^{+}\right)$$

where *G*(anion) is the total Gibbs free energy of the deprotonated anion and *G*(enol) is the total Gibbs free energy of the parent molecule in enol form. In the case of prototropic tautomerisation, the reaction Gibbs free energy Δ_r_ *G* ^T^ (tau) represents the difference

3 $$\Delta_{r}G^{T}={G^{{}^{\circ}}}_{1M}\left(\mathrm{keto}\right)-{G^{{}^{\circ}}}_{1M}\left(\mathrm{enol}\right)$$

where the total Gibbs free energy of the keto form is denoted by *G*(keto).

The ChemSketch^30^ and Avogadro programs^31^ were used for drawing the studied species and visualization of optimal geometries, respectively.

## 3 Results and Discussion

In accordance with previously published experimental and theoretical works, the optimal geometries of the studied *trans*-stilbenols in the ground electronic state are planar. In the case of compounds **35_345** and **345_345**, the mutual orientation of hydroxy groups is determined by intramolecular hydrogen bonds. This planar conformation exerts a substantial influence on the structural stability and the physicochemical properties of these compounds. The identified optimal geometries of the molecules in the ground electronic state and in the enol form are shown in Figure S1.

The ability to abstract a proton from the −OH group and to form the corresponding anion can be estimated from the corresponding reaction Gibbs free energy (Δ_r_ *G*) calculated according to eq 2. For example, the proton abstraction from the hydroxy group of the reference phenol molecule requires, in aqueous solution, an energy of 99 kJ mol^–1^. This energy can decrease after the addition of a second phenyl ring. For example, the Δ_r_ *G* is 93 and 96 kJ mol^–1^ for the reference 1-naphthol and 2-naphthol, respectively. The calculated reaction free energies can be related to the acidity constants, usually presented as p*K*_a_ values

4 $$pK_{a}=\Delta_{r}G/\left[RT\;\ln\left(10\right)\right]$$

where *R* is the universal gas constant. The obtained theoretical p*K*_a_ value for reference phenol in water is 17.3, while the experimental value is 9.8.^11^ Similar discrepancies were found for the reference molecules, i.e., 1-naphthol and 2-naphthol (see Table S1). The origin of this discrepancy likely stems from the insufficient description of solvent effects and the theoretical limits of the implicit solvation model. A mutual comparison of p*K*_a_ values for the first deprotonation step (see Table 1) reveals the endergonic character of this process. In the case of monosubstituted compounds, the p*K*_a_ values vary depending on the position of the hydroxy group. The lowest value of 16.1 is for the *para* position (our notation is **4**), followed by the *ortho* positions (16.3 and 16.5), and finally the *meta* position with values of 18.1 and 17.1. For more substituted molecules, the minimal p*K*_a_ value of 13.6 is exhibited by the hexa-substituted molecule **345_345**, where the hydroxy group in the 4́ position is deprotonated. Deprotonation of the −OH group at position 4 requires a slightly higher energy because the p*K*_a_ is 12.4. The maximal value of 17.3 belongs to the trisubstituted molecule **35_4,** where the proton is abstracted from the −OH group in position No. 5. In general, it appears that OH groups in the para position exhibit a more acidic character than the remaining hydroxy groups. The exception is represented by the molecule **24_4** (see Table 1).

**Table 1:** Calculated p*K*_a_ (p*K*_a_*) Values^a^

**Molecule**	**Anion 1**	**Anion 2**	**Anion 3**	**Anion 4**	**Anion 5**	**Anion 6**
**2**	16.3 (8.8)					
**3**	18.1 (10.9)					
**4**	16.1 (9.8)					
**5**	17.1 (12.0)					
**6**	16.5 (8.0)					
**24**	15.1 (9.2)	15.4 (10.3)				
**35**	17.4 (10.0)	17.8 (10.3)				
**24_4**	15.8 (11.2)	16.5 (11.7)	17.3 (12.7)			
**35_2**	17.9 (11.0)	17.9 (11.8)	16.0 (7.7)			
**35_4**	17.3 (14.0)	18.7 (15.6)	16.5 (10.7)			
**35_5**	15.1 (10.2)	16.5 (9.9)	17.1 (11.2)			
**35_6**	17.4 (11.1)	17.7 (12.0)	15.0 (9.3)			
**35_35**	16.6 (11.3)	16.5 (12.3)	16.3 (10.9)	16.5 (12.4)		
**35_345**	17.2 (14.2)	17.5 (14.1)	14.8 (8.8)	14.1 (6.2)	16.3 (11.0)	
**345_345**	14.5 (10.0)	13.8 (7.5)	16.9 (13.3)	16.9 (12.6)	13.6 (7.6)	14.3 (11.0)

a The values for deprotonated monoanions are listed in the order of the numbering of OH groups indicated in molecular notation. The data for the lowest excited state are in parentheses. These values were calculated according to the eq 4.

As shown in Figure S2, the dependence of available experimental p*K*_a‑exp_ values in water for reference phenol, 1-naphthol, 2-naphthol,^11^ and 4-hydroxystilbenol^5^ on the calculated p*K*_a_ is linear.

5 $$pK_{a,exp}=0.401\times pK_{a}+2.85$$

The obtained correlation coefficient was 0.993. This linear dependence has a nonzero intercept, reflecting the imperfections and simplifications of the quantum chemical model employed.

The scaled theoretical p*K*_a,sc_ quantities obtained using eq 5 are depicted in Figure 2.

These values also show the same trends in increase and decrease as demonstrated for the calculated p*K*_a_’s. The values range from 8.3 for **345_345** to 10.3 for **35_4**. It should be noted that experimental p*K*_a,exp_ values may differ depending on the measurement method used. For example, the published p*K*_a1,exp_ values for resveratrol in solvents with different water contents vary between 8.0 and 10.3. Our theoretical value of 9.5 is in good agreement with the experimental value of 9.4 measured by capillary zone electrophoresis.^32^

In general, the calculation of optimal geometries and thermodynamic quantities of molecules in electronic excited states is not a trivial problem. This is because the DFT approach itself and the corresponding functionals were formulated for the ground state. Since the molecules studied are planar even in the S_1_ state, the contributions of the vibrational and rotational motions of the molecule to the Gibbs energy will differ minimally from those in the electronic ground state. However, the calculation of the de-excitation energy corresponding to the fluorescence energy will play a key role. For organic molecules with an aromatic structure, the B3LYP functional appears to be the optimal choice. The calculated p*K*_a_* for the protolytic reaction, where the enol form and corresponding monoanion are in the lowest excited state S_1_ are summarized in Table 1 (see values in parentheses). Compared with the electronic ground state, all p*K*_a_* values exhibit lower values, indicating the presence of photoacidity. The values for monosubstituted derivatives range from 8.0 for **6** to 12.0 for **5** (Table 1). This interval is two times smaller than for the monosubstituted molecules in the electronic ground state. For more substituted derivatives, the p*K*_a_* interval ranges from 6.2 for **35_345** to 15.6 for **35_4**. This interval is also smaller than in the ground state. The mutual comparison of calculated p*K*_a_* values also indicates a strong dependence on the deprotonation site. It seems that the formation of an O-anion located at the C3/C3' atom from molecules with substituted C3/C3' and C5/C5' positions relates to the largest p*K*_a_* value. In connection with the use of the TD-DFT/B3LYP scheme, problems associated with the description of intramolecular charge transfer states may arise. This problem is particularly pronounced in stilbenes substituted with pairs of strong electron donors and strong electron acceptors. The presence of intramolecular charge transfer is accompanied by an artifact where a low-energy transition with zero oscillator strength appears. In our case, a low oscillator strength value appeared for de-excitations of systems that were deprotonated or involved prototropic tautomerism (see next text). Analysis of the molecular orbitals predominantly participating in the respective type of excitation demonstrated a clear π*−π character (Figure S4). The low oscillator strength in these cases indicates short-lived fluorescent states.

The measurements of p*K*_a_* constants for electronically excited states are much more challenging than for the electronic ground state. The p*K*_a_* of the excited states are determined by various spectroscopic methods. The most popular method is based on the so-called Förster cycle^33^ which can be considered a thermodynamic method. The attainment of protolytic equilibrium in the excited state is not necessary in this method; it is sufficient to know the spectroscopic characteristics of the starting materials and the final products of the photoprotolytic dissociation. The alternative methods are based on the measurement of the intensity or quantum yields of the fluorescence under steady-state conditions.^34^ The difference between p*K*_a_* values for the ground and excited states can reach several orders of magnitude. Furthermore, the p*K*_a_* values measured using different methods can vary significantly. To the best of our knowledge, only two experimental data points regarding the p*K*_a_* values of stilbenols are currently available in the scientific literature, and these are reported to have low measurement uncertainty. Notwithstanding, an alternative approach employing scaled values is presented in Figure S5.

Isomerization of the enol tautomer to possible keto tautomers requires the disruption of aromaticity and is, therefore, strongly endergonic (Δ*G* > 0). The reaction Gibbs free energies of tautomerization are calculated as the difference between the Gibbs free energies of enol and keto forms according to eq 3.

In the case of a simple phenol molecule, the corresponding enol tautomer is the thermodynamically more stable molecule, featuring effective π-electron delocalization. The formation of 2,4-cyclohexadienone and 2,5-cyclohexadienone requires reaction Gibbs free energy of 59 and 64 kJ mol^–1^, respectively. Like the acid–base equilibrium, the p*K*_T_ constant can be calculated as

6 $$pK_{T}=\left[G{{\;}^{{}^{\circ}}}_{1M}\left(\mathrm{keto}\right)-G{{\;}^{{}^{\circ}}}_{1M}\left(\mathrm{enol}\right)\right]/\left(RT\;\ln10\right)$$

The corresponding values for cyclohexadienones were 10.3 and 11.3, respectively. For 1-naphthol, the added aromatic ring decreases the p*K*_T_ to values of 4.9 for 2,4-benzocyclohexadienone and 5.4 for 2,5-benzocyclohexadienone (see Figure S3 and Table S2). The possible enol and ketotautomers in **2**-, **3**-, and **4**-monostilbenols are shown in Figure 3.

**Figure 3:**
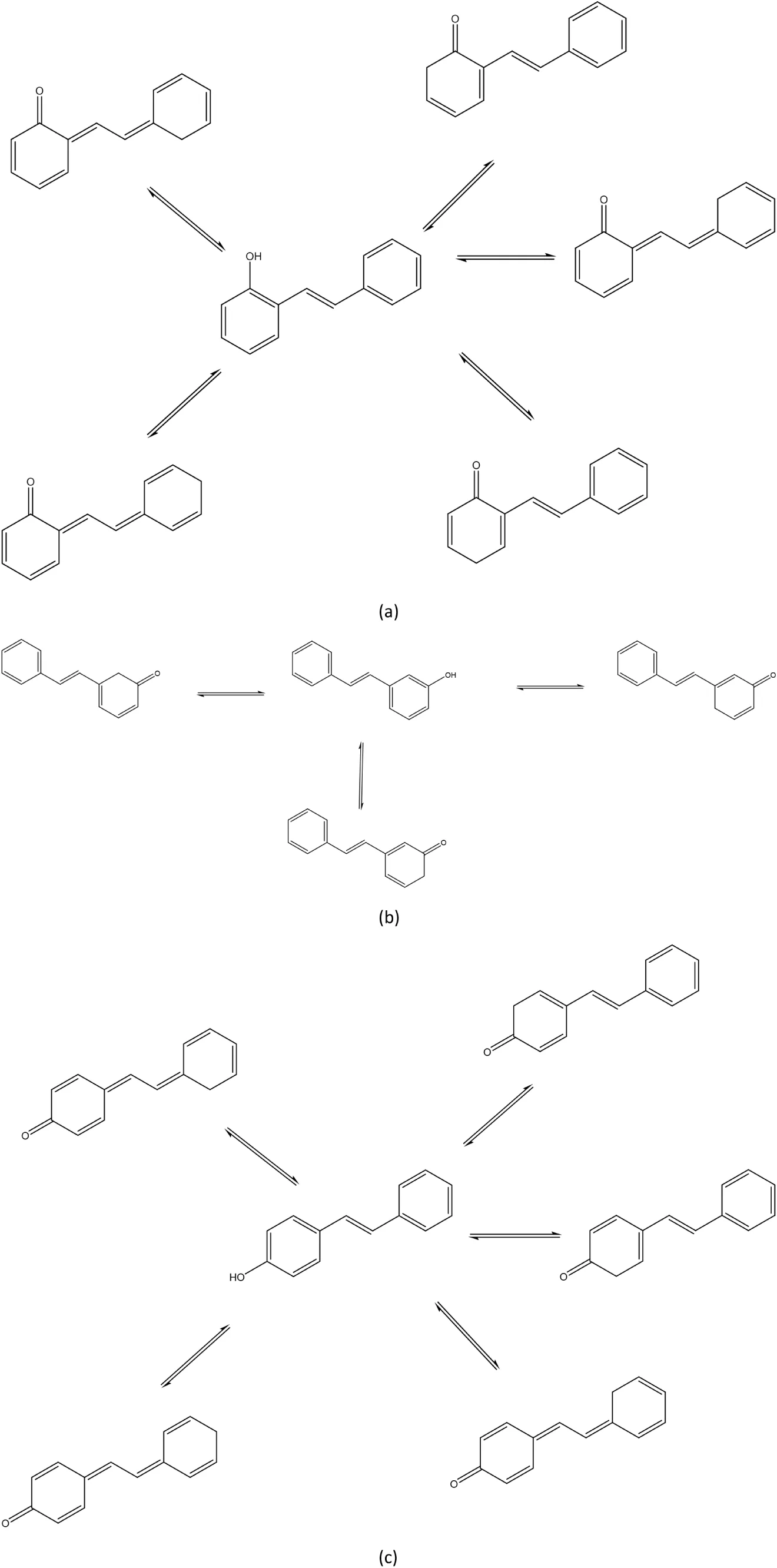
Possible enol and keto tautomers occurring in monostilbenols: **2** (a), **3** (b), and **4** (c).

As can be seen in Table 2, the p*K*_T_ for the monosubstituted derivative **3** has the lowest value of 8.1. The proton moves from the OH group to position C6. Similar p*K*_T_ value of 8.4 has derivative **5** where the proton transfers to C6. The possible formation of tautomers in compounds **2** and **6** on the second aromatic ring is thermodynamically unfavorable. The calculated p*K*_T_ values are higher than 20.7. The presence of multiple hydroxy groups significantly affects C–H tautomerism. The derivative **35_35** has the lowest p*K*_T_ values of 3.5 and 3.7 (see Table 3). A proton migrates from the OH group on carbon 3’ to carbon 6’ and or from the OH group on carbon 5’ to carbon 6’. Similar results were also found for **345_345**.

**Table 2:** Calculated p*K*_T_ Constants for the Prototropic Equilibrium between Hydroxy Group and C-Protonated Atoms for Mono- and Bisubstituted Molecules^a^

	**2**	**3**	**4**	**5**	**6**	**24**	**35**
**C2=O**							
**C2'H**_**2**_	23.3 (1.7)					20.5 (1.8)	
**C3H**_**2**_	11.3 (1.5)					8.5 (−2.0)	
**C4'H**_**2**_	20.9 (1.0)					18.0 (1.1)	
**C5H**_**2**_	10.8 (10.5)					7.1 (8.4)	
**C6'H**_**2**_	22.7 (−0.1)					19.6 (−0.3)	
**C3=O**							
**C2H**_**2**_		9.7 (−2.8)					6.6 (−4.7)
**C4H**_**2**_		9.2 (3.5)					7.5 (−0.8)
**C6H**_**2**_		8.1 (3.1)					5.1 (−0.9)
**C4=O**							
**C2'H**_**2**_			21.7 (2.3)				
**C3H**_**2**_			11.5 (4.8)			8.7 (1.8)	
**C4'H**_**2**_			19.2 (2.8)				
**C5H**_**2**_			12.3 (5.0)			8.5 (6.4)	
**C6'H**_**2**_			20.9 (0.6)				
**C5=O**							
**C2H**_**2**_				9.2 (4.6)			6.9 (0.6)
**C4H**_**2**_				10.2 (2.2)			8.0 (−2.7)
**C6H**_**2**_				8.4 (−3.5)			5.4 (−5.4)
**C6=O**							
**C2'H**_**2**_					22.5 (−2.8)		
**C3H**_**2**_					9.9 (6.7)		
**C4'H**_**2**_					20.2 (−3.7)		
**C5H**_**2**_					11.0 (−1.7)		
**C6'H**_**2**_					22.0 (−4.2)		

a The p*K*_T_* values for the electronic excited state S_1_ are written in parentheses.

**Table 3:** Calculated p*K*_T_ Constants for the Prototropic Equilibrium between Hydroxy Group and C-Protonated Atoms for More Substituted Molecules^a^

	**24_4**	**35_2**	**35_4**	**35_5**	**35_6**	**35_35**	**35_345**	**345_345**
**C2=O**								
**C2'H**_**2**_	21.9 (2.2)							
**C3H**_**2**_	8.1 (−3.1)							
**C4'H**_**2**_								
**C5H**_**2**_	7.7 (6.6)							
**C6'H**_**2**_	20.9 (0.0)							
**C2'=O**								
**C2H**_**2**_		15.8 (−4.6)						
**C4H**_**2**_		15.3 (−4.5)						
**C6H**_**2**_		14.9 (−6.5)						
**C3=O**								
**C2H**_**2**_		6.9 (−4.0)	6.4 (−3.3)	5.4 (−5.4)	6.3 (−5.0)	5.6 (−3.0)	6.9 (−3.8)	5.5 (−6.9)
**C4H**_**2**_		7.5 (0.3)	6.8 (0.2)	5.8 (−1.8)	6.9 (−0.6)	5.7 (−0.1)		
**C6H**_**2**_		5.3 (−0.8)	5.6 (−0.1)		4.3 (−1.7)	4.4 (0.4)	5.4 (−1.3)	3.8 (−5.3)
**C3'=O**								
**C2'H**_**2**_						5.3 (−2.9)	6.4 (−6.0)	7.5 (−4.7)
**C4'H**_**2**_						5.9 (0.7)		
**C6'H**_**2**_						3.5 (0.3)	5.7 (−3.3)	5.7 (−1.9)
**C4=O**								
**C2'H**_**2**_								14.1 (−6.6)
**C3H**_**2**_	8.7 (−0.0)							
**C4'H**_**2**_								
**C5H**_**2**_	8.7 (4.9)							
**C6'H**_**2**_	20.2 (1.3)							13.5 (−8.1)
**C4'=O**								
**C2H**_**2**_			15.7 (−2.1)				13.5 (−7.0)	13.8 (−6.2)
**C3'H**_**2**_			11.9 (5.9)					
**C4H**_**2**_							11.6 (−7.5)	
**C5'H**_**2**_			13.2 (6.4)					
**C6H**_**2**_	21.9 (1.3)		14.7 (−4.4)				12.6 (−9.3)	13.8 (−8.7)
**C5=O**								
**C2H**_**2**_		6.2 (−0.3)	6.8 (0.6)	5.9 (−0.3)	6.6 (−0.2)	5.6 (1.7)	7.0 (0.2)	7.2 (−0.6)
**C4H**_**2**_		8.0 (−1.6)	7.7 (−1.2)	6.5 (−3.5)	7.6 (−2.5)	6.6 (−1.3)		
**C6H**_**2**_		5.4 (−5.0)	4.9 (−5.0)		5.0 (−5.8)	4.0 (−3.9)	5.0 (−5.0)	5.5 (−7.0)
**C5' = O**								
**C2'H**_**2**_				8.4 (3.4)		5.6 (1.8)	8.1 (0.7)	5.2 (−3.7)
**C4'H**_**2**_				9.6 (1.1)		6.4 (−1.2)		
**C6'H**_**2**_				7.9 (−4.2)		3.7 (−3.9)	5.9 (−5.6)	3.9 (−8.6)
**C6=O**								
**C2'H**_**2**_								
**C3H**_**2**_								
**C4'H**_**2**_								
**C5H**_**2**_								
**C6'H**_**2**_								
**C6’=O**								
**C2H**_**2**_					15.4 (−6.4)			
**C3'H**_**2**_					8.9 (8.3)			
**C4H**_**2**_					14.3 (−6.4)			
**C5'H**_**2**_					9.4 (0.0)			
**C6H**_**2**_					14.0 (−8.1)			

a The p*K*_T_* values for the electronic excited state S_1_ are written in parentheses.

In the case of the tautomeric equilibrium studied, four experimental p*K*_T,exp_ constants for phenol and 1-naphthol (see Table S1) are available in the literature for room temperature in acidic water solution. The correlation of available experimental p*K*_T,exp_ values for phenol and 1-naphthol with the corresponding p*K*_T_ leads to the linear function

7 $$pK_{T,exp}=1.79+0.936\times pK_{T}$$

with coefficient of determination *R* ^2^ = 0.986 (Figure S3 and Table S2). Quantitatively, this finding suggests that the Gibbs free energy of reaction (Δ_r_ *G* ^T^) has been underestimated. This discrepancy is likely due to the dissolution corrections, which depend on the specific chemical structures of the keto and enol tautomers. Consequently, using eq 7, we can calculate the scaled equilibrium constants (p*K*_T,sc_) for the possible tautomers of the studied molecules. These results are collected in Tables S3 and S4 and the most thermodynamically preferred p*K*_T,sc_ values are depicted in Figure 2.

These scaled p*K*_T,sc_ values exhibit similar trends with respect to chemical structure. The range of values extends from 5.1 for **35_35** to 23.6 for **2**. The largest p*K*_T,sc_ values are associated with tautomeric equilibria between the terminal aromatic rings. Figure 2 provides a comparative analysis of the influence of the chemical structure of the studied molecules on the minimum p*K*_T,sc_ values. All these values are positive, indicating very low to negligible concentrations of keto tautomers in the actual sample. Values in the range of 5.1 to 5.5 are exhibited only by molecules **35_35** and **345_345**, where the tautomeric equilibrium occurs between the C3/C3'–OH and C6/C6' positions.

The calculated p*K*_T_* values are summarized in Tables 2 and 3. The range of these values extends from a negative value of −9.3 for **35_345** to 10.5 for **2**. The negative values indicate the exergonicity of this internal proton transfer and a clear shift in the tautomeric equilibrium toward the keto form. Nevertheless, it is important to note that other factors may also influence the process under study. For example, the lifetime of the excited state, intersystem crossing to triplet states, and vibrational relaxation and reorganization of solvent molecules. While the values within this framework provide a qualitative ranking of excited-state tautomeric stability, it is worth keeping in mind that TD-DFT calculations do not explicitly include the nonadiabatic coupling involved in excited-state proton transfer processes. Therefore, these values are best interpreted as a measure of thermodynamic propensity rather than absolute quantitative predictions.

## 4 Conclusions

A systematic theoretical study of 15 naturally occurring *trans*-stilbenols in water was investigated using density functional theory. The thermodynamics of protonation equilibria and the tautomeric equilibria of enol and keto-tautomers were evaluated. The acidity constants (p*K*_a_) and tautomerization constants (*pK*_T_) were estimated for the electronic ground (S_0_) and lowest excited (S_1_) states. Theoretical calculations demonstrated the strong influence and synergistic effects of hydroxy group position(s) on the studied equilibria. After optical excitation, the molecules studied exhibit not only enhanced thermodynamic acidity but also greater thermodynamic stability of keto-tautomers relative to the parent enol forms. Although we encountered limitations in the method, both in terms of solvation and the single-determinant approximation when calculating thermodynamic quantities for molecules in the excited state (S^1^), the trends obtained can be considered qualitatively adequate. The negative values of the reaction Gibbs energy at tautomeric equilibria indicate that the keto form may be populated in the excited state. Furthermore, the feasibility of proton transport from a hydroxy group located on one aromatic ring to a neighboring ring along the −CH=CH– bridge was also theoretically demonstrated. These findings may play an important role in de-excitation processes as well as in various biochemical defense mechanisms of plants.

## Supplementary Material



## Data Availability

The data that support the findings of this study are available in the article’s Supporting Information.

## Notes

B3LYP/6–311++G­(d,p) optimal geometries and Gibbs free energies (Figure S1); correlation between experimental p*K*_a_ values and B3LYP­(water)/6–311++G­(d,p) p*K*_a,cal_ (Figure S2); correlation between experimental p*K*_T_ values and theoretical p*K*_T, cal_ and tautomerism reactions (Figure S3); frontier molecular orbitals of the first electron transition in the first excited state (Figure S4); the p*K**_a,sc_ values and the scaled p*K*_T,sc_ values for energetically preferred keto–enol tautomerisation equilibria (Figure S5); calculated reaction Gibbs free energies, theoretical p*K*_a_ values, and experimental p*K*_a,exp_ values (Table S1); calculated reaction Gibbs free energies, theoretical p*K*_T,_ and experimental p*K*_T,exp_ values (Table S2); scaled p*K*_T,sc_ constants for the prototropic equilibrium between the hydroxy group and C-protonated atoms for for mono- and bisubstituted molecules (Table S3) and more substituted molecules (Table S4) (PDF)
