# Supplementary material for: Thermodynamics of Acidity and Photoacidity in trans-Resveratrol Analogues: The Role of Prototropic Tautomerism
Source: ACS Omega. 2026 Aug 8;11(33):50357–65. doi: 10.1021/acsomega.6c06306 (PMC13520537; doi:10.1021/acsomega.6c06306)
Supplement: Supplementary file 1 [file ao6c06306_si_001.pdf]

On the Thermodynamics of Acidity and Photoacidity in *trans*-Resveratrol Analogues: The Role of Prototropic Tautomerism

Andrea Kováčová [a)*], Martin Michalík [a)], Vladimír Lukeš [a)]

[a)] Faculty of Chemical and Food Technology, Slovak University of Technology in Bratislava, Radlinského 9, SK-812 37 Bratislava, Slovakia

* Corresponding author: andrea.kovacova@stuba.sk

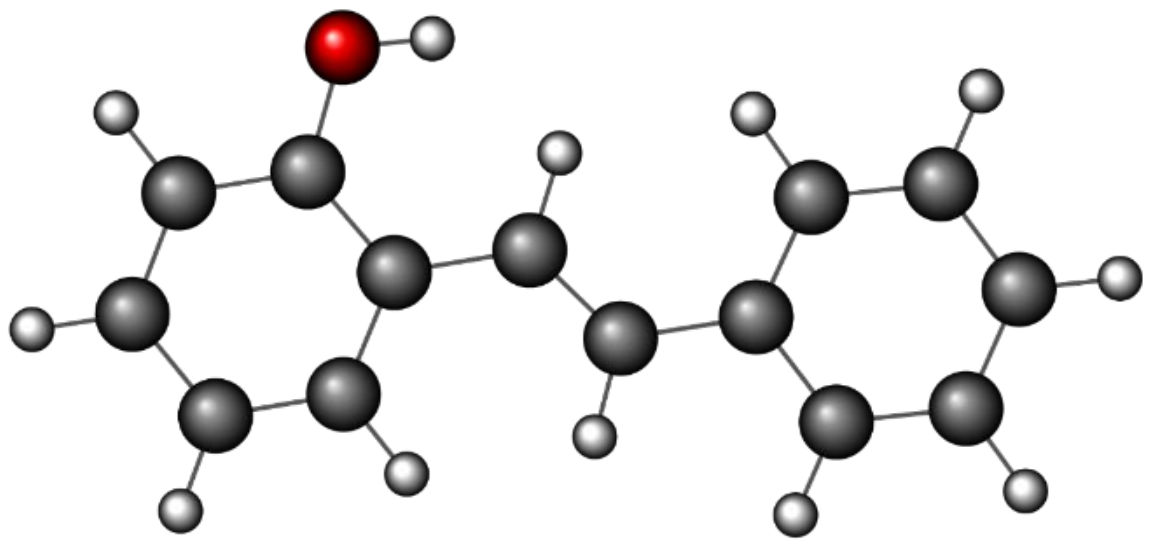

2

G = –615.928377 Hartree

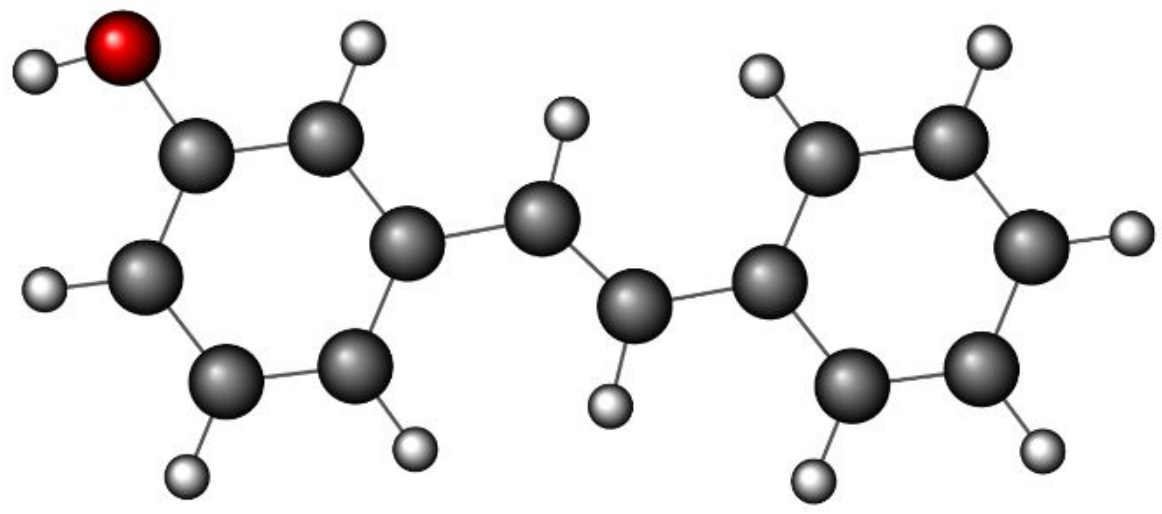

3

G = –615.929468 Hartree

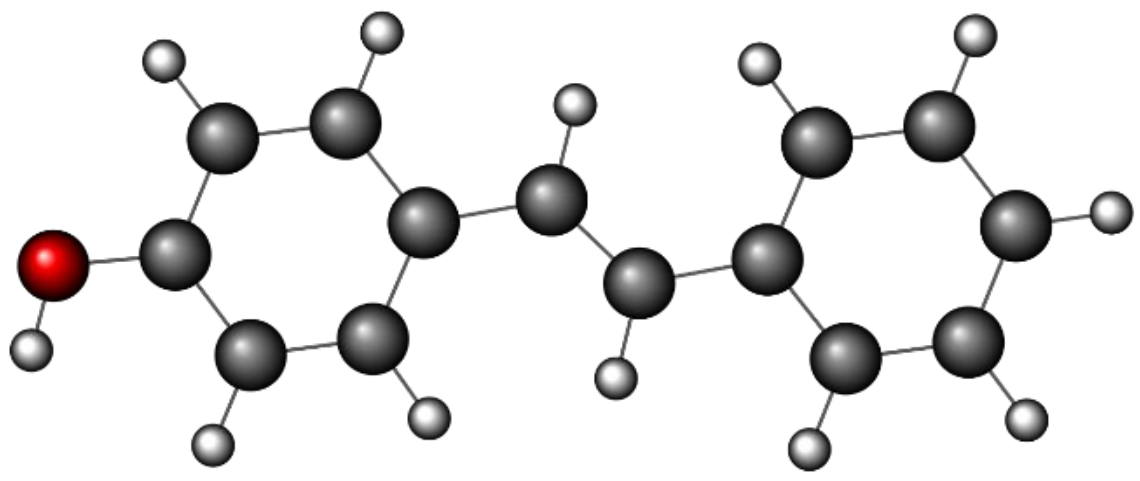

4

G = –615.929783 Hartree

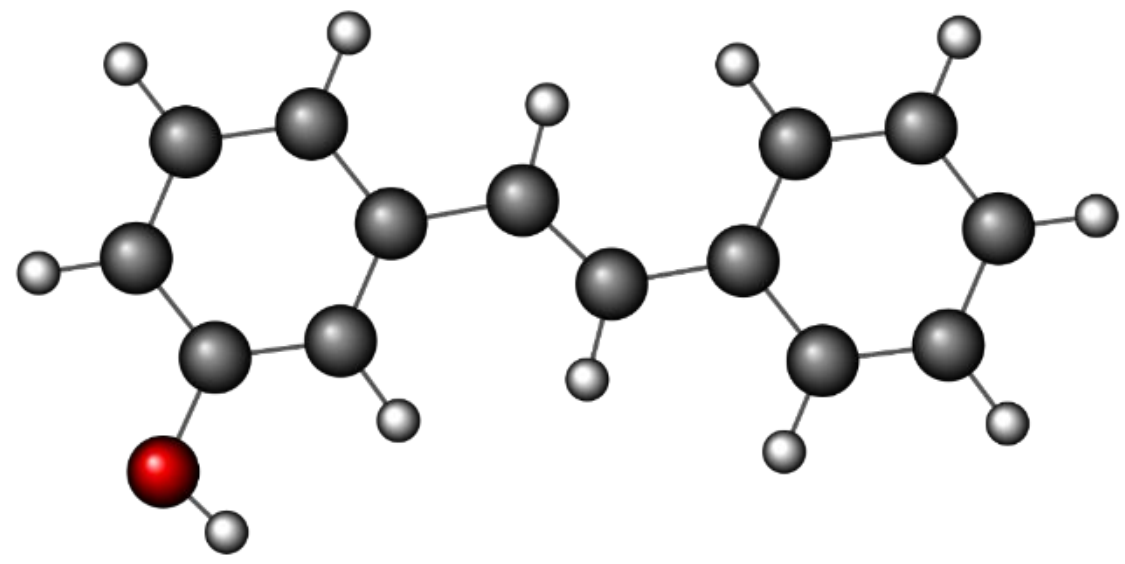

5

G = –615.929317 Hartree

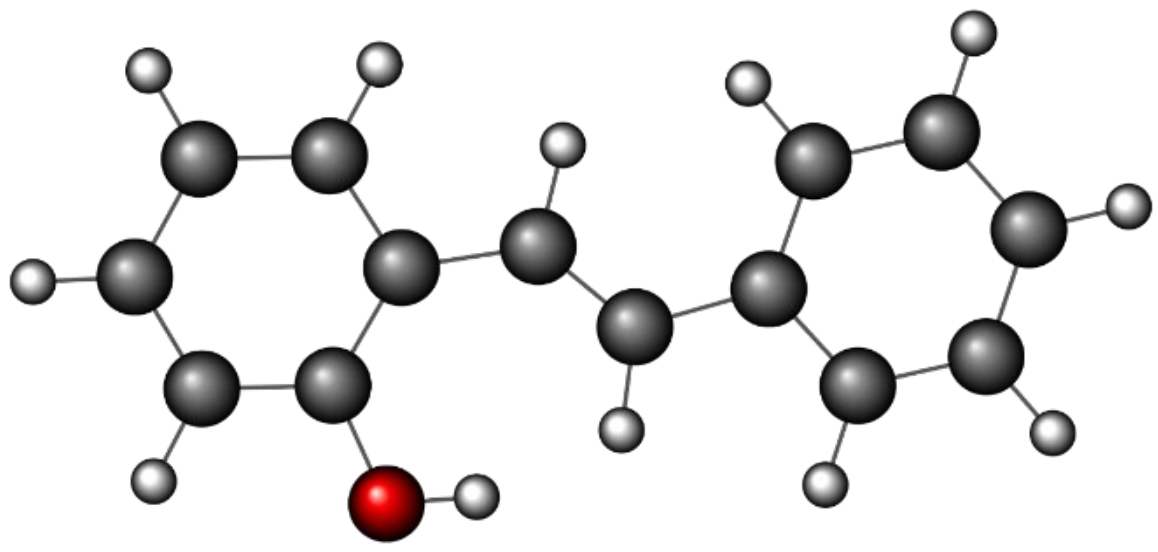

6

G = –615.925896 Hartree

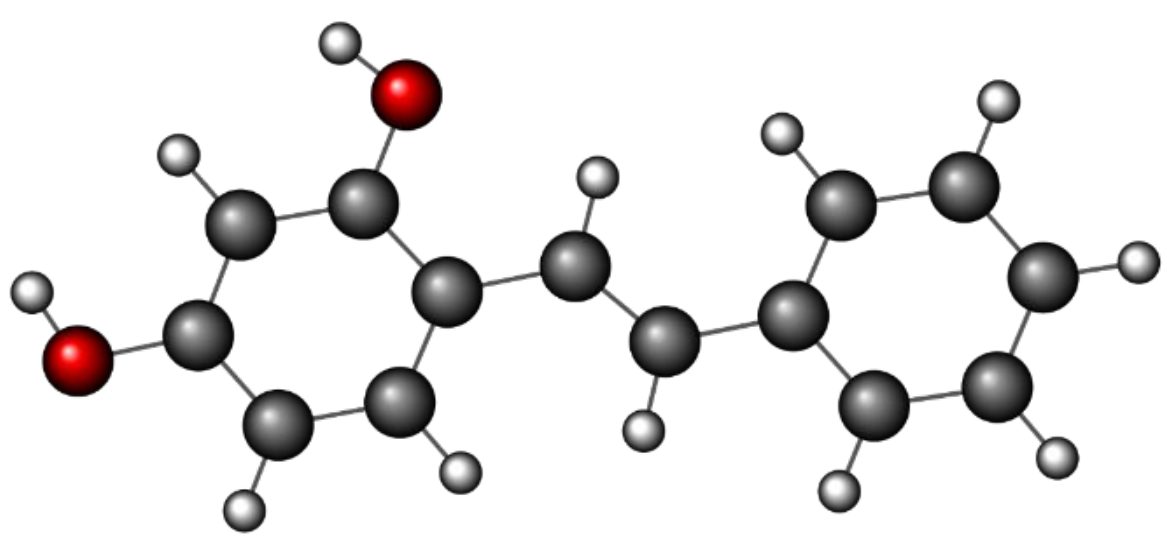

24

G = –691.180227 Hartree

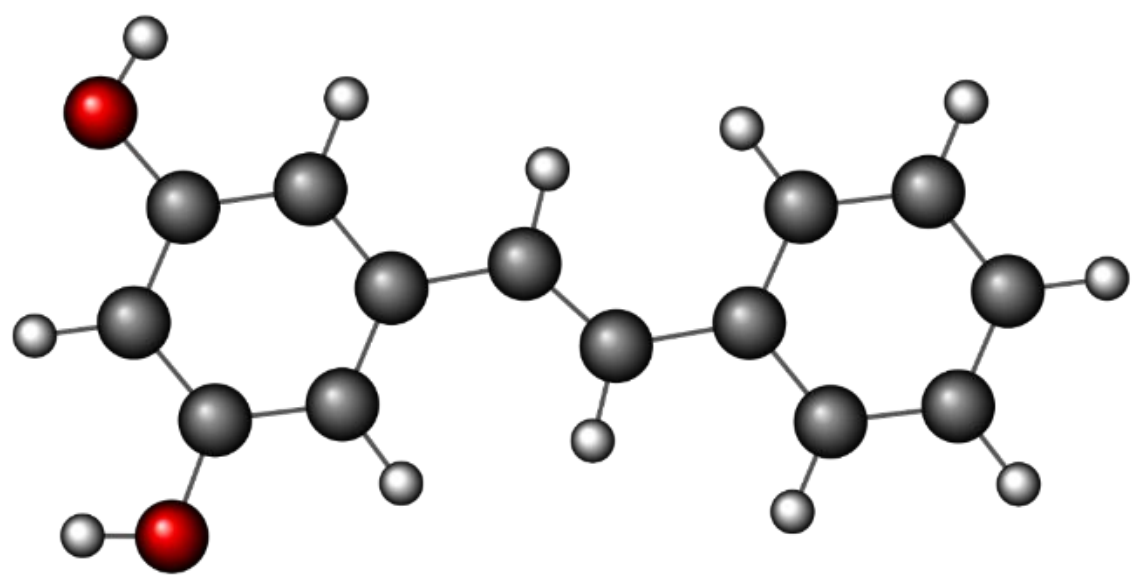

35

G = –691.183155 Hartree

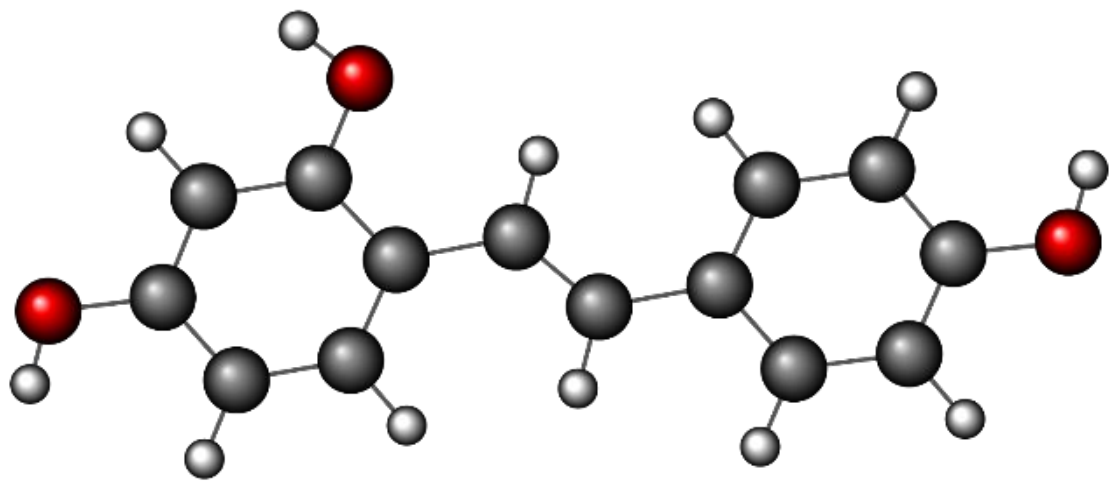

24_4

G = –766.433903 Hartree

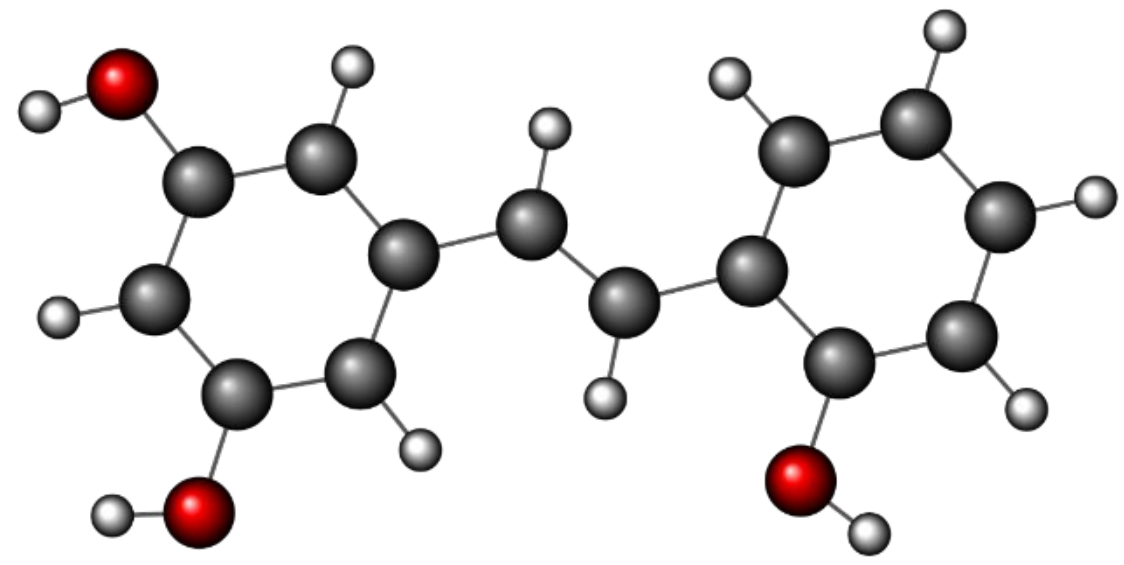

35_2

G = –766.433876 Hartree

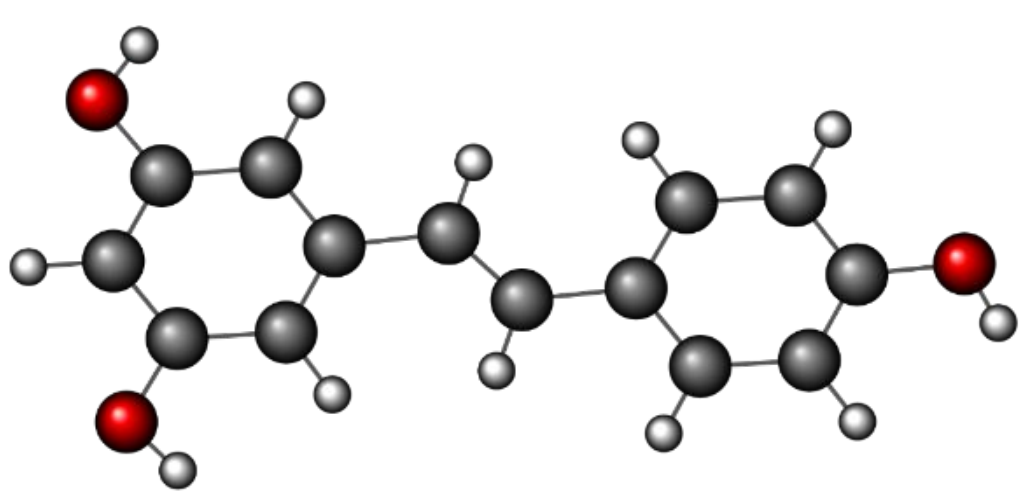

35_4

G = –766.437397 Hartree

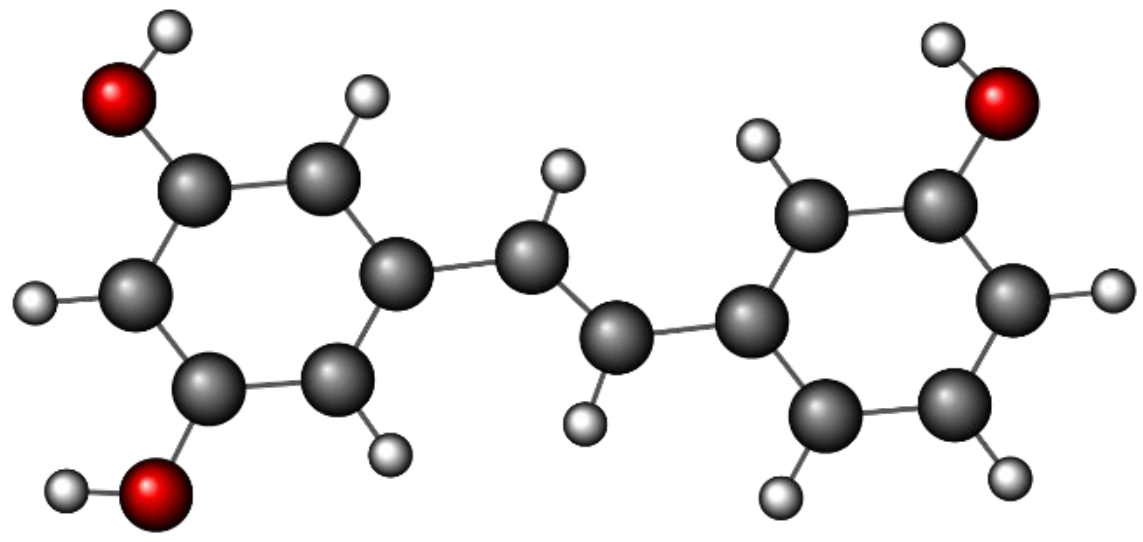

35_5

G = –766.433104 Hartree

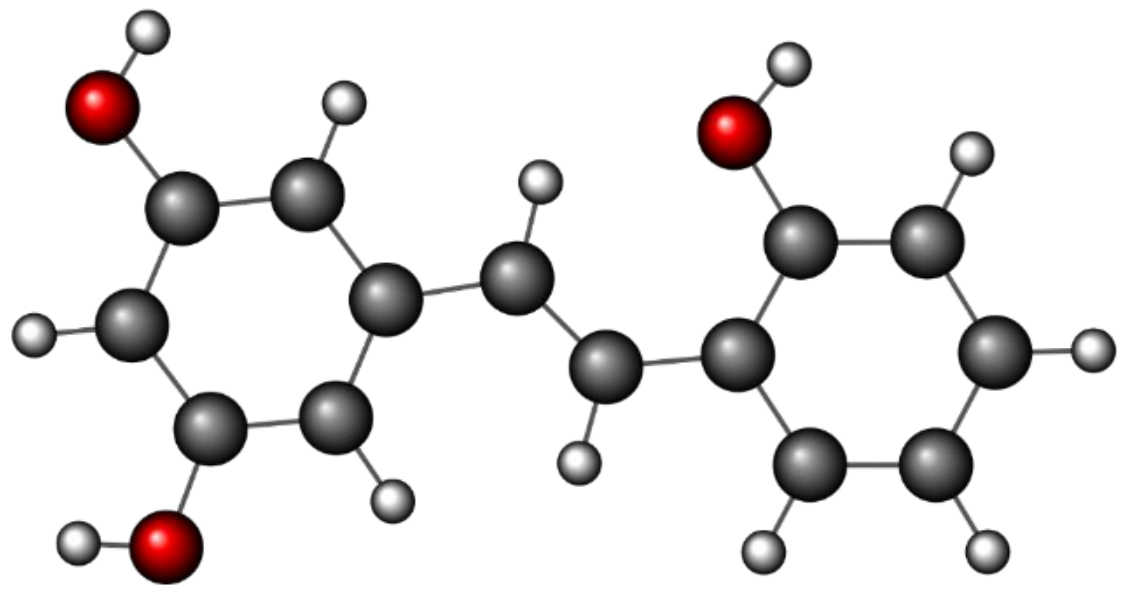

35_6

G = –766.431498 Hartree

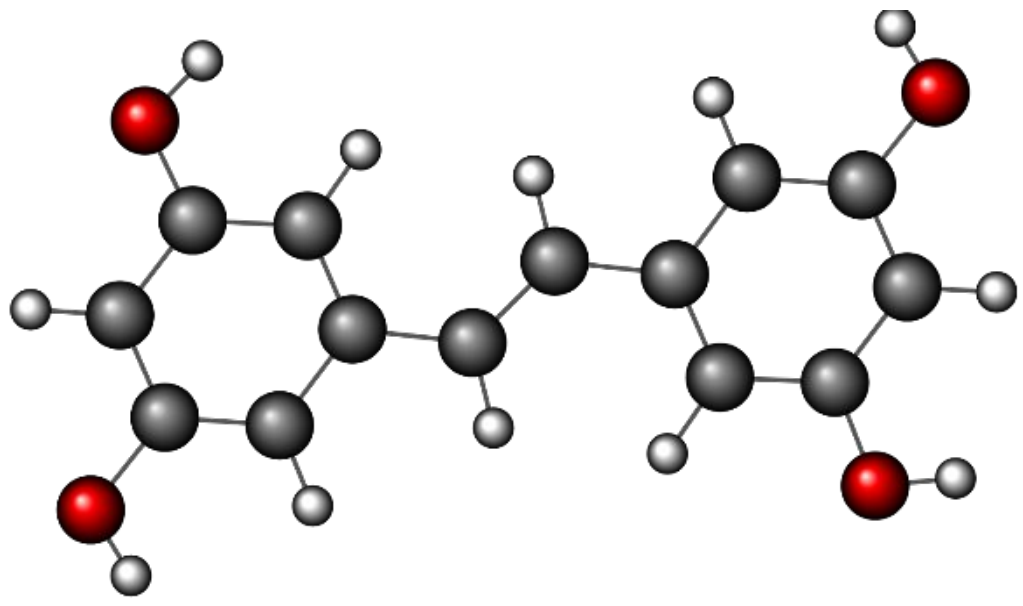

35_35

G = –841.686293 Hartree

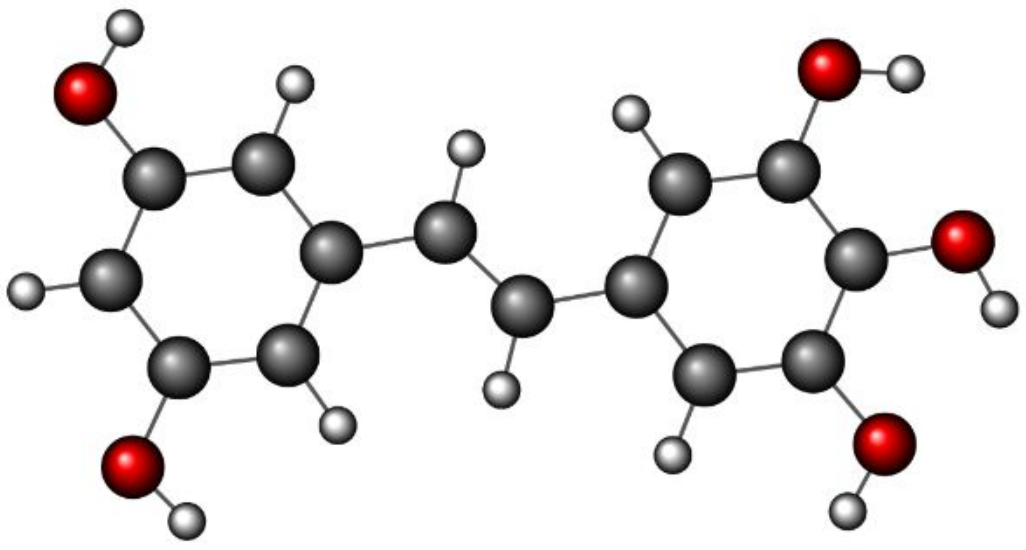

35_345

G = –916.841193 Hartree

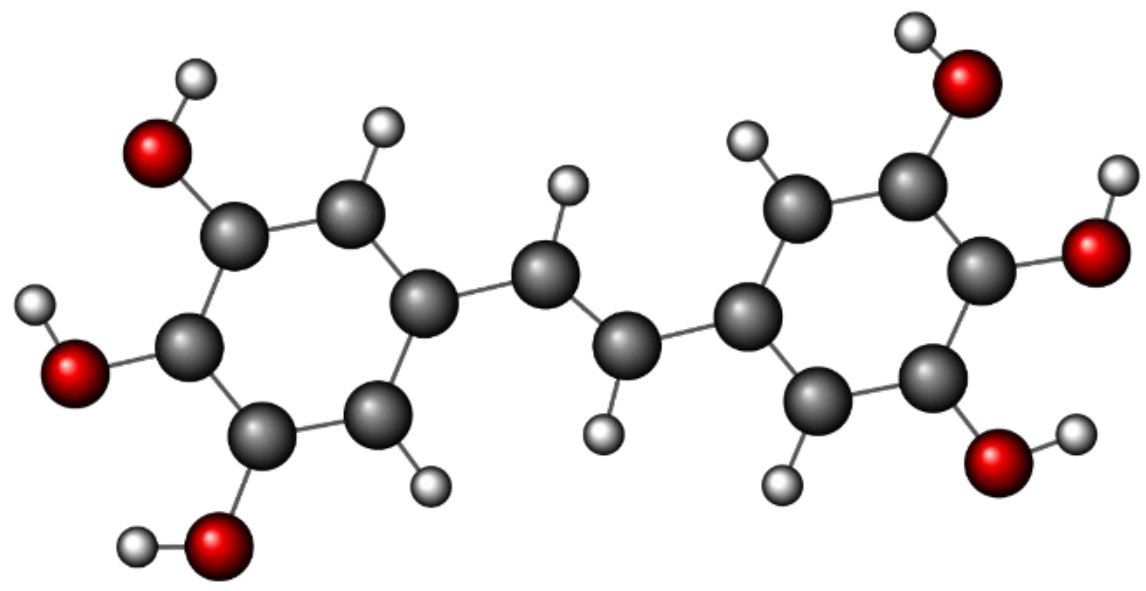

345_345

G = –992.182129 Hartree

Figure S1. Calculated B3LYP/6-311++G(d,p) optimal geometries and Gibbs free energies (hartree) for studied molecules in neutral form of electronic ground state.

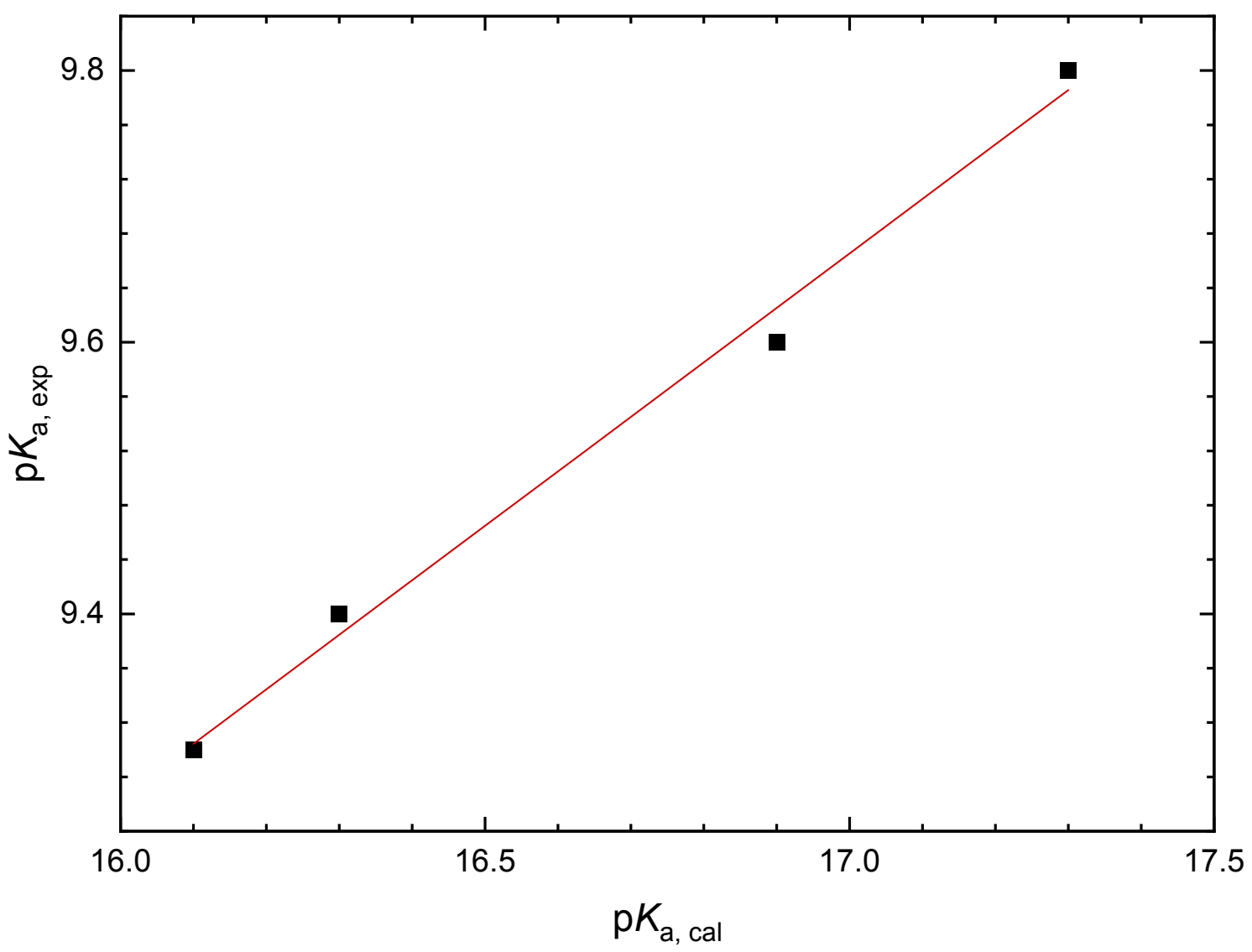

Figure S2. Correlation between available experimental pK_a values and calculated B3LYP(water)/6-311++G(d,p) p$K_{a,cal}$ for reference phenol, 1-naphthol, 2-naphthol and 4-stilbenol (**4**). Intercept is 2.85 ± 0.41 and slope 0.401 ± 0.025 with R^2 = 0.993.

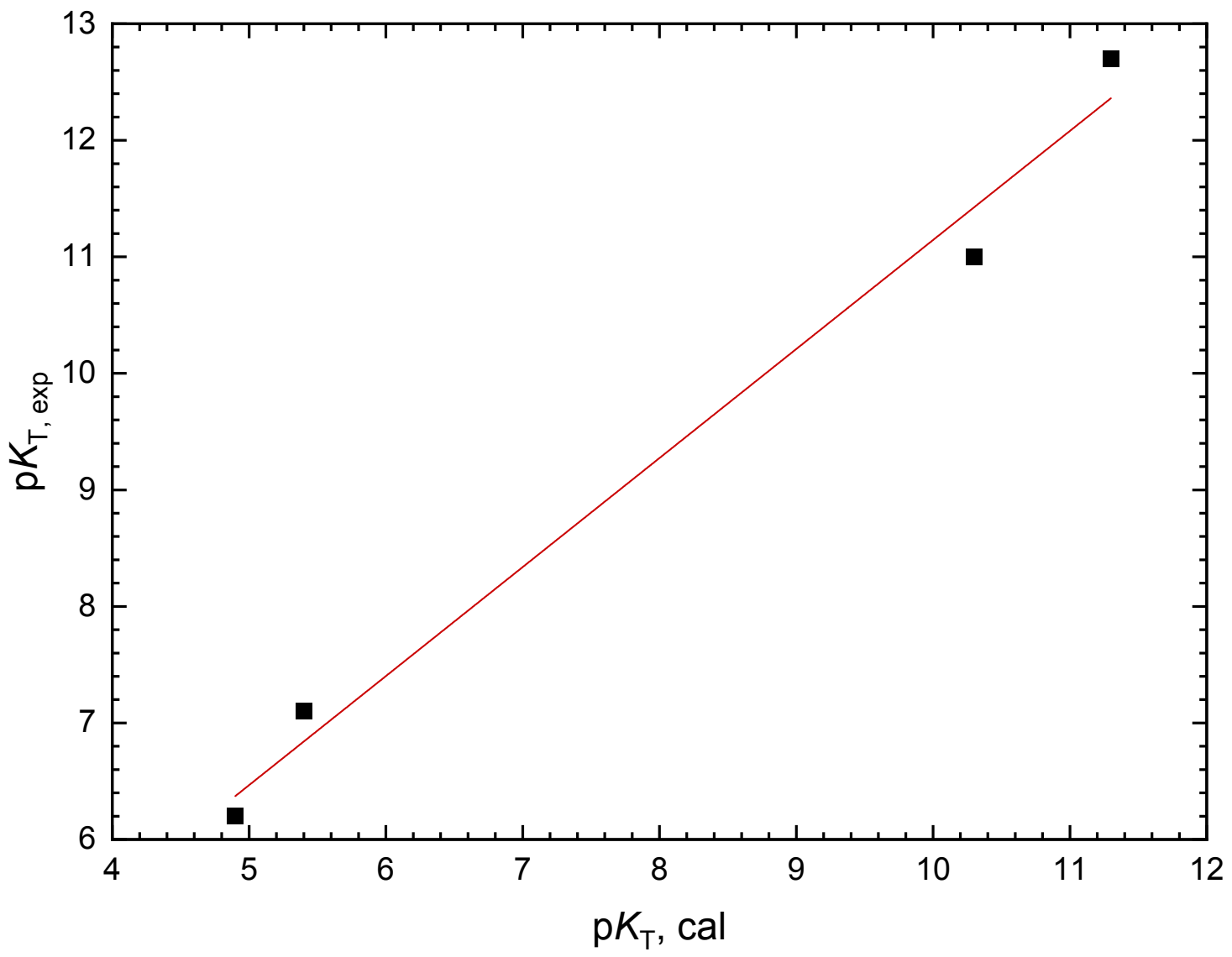

a)

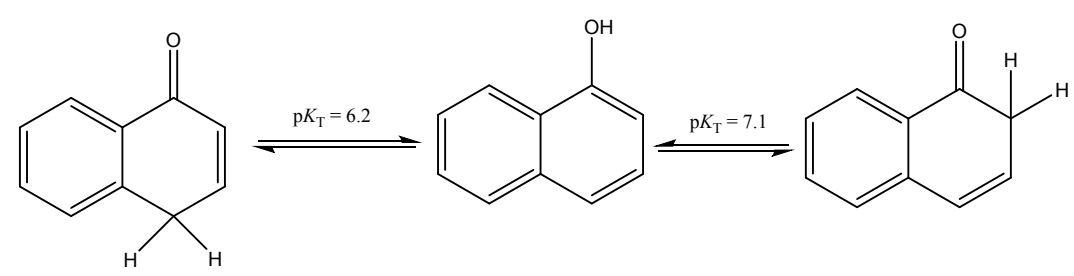

b)

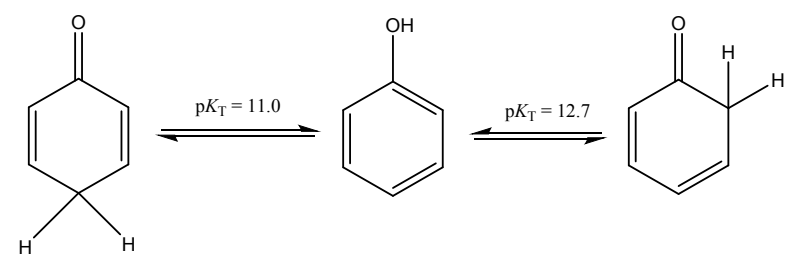

c)

Figure S3. a) Correlation between available experimental pK_T values and calculation B3LYP(water)/6-311++G(d,p) p$K_{T,\ cal}$ of studied reaction. Intercept is 1.79 ± 0.66 and slope 0.936 ± 0.078 with R^2 = 0.979. The studied tautomerism reaction and the available experimental pK_T values in water for b) naphthalen-1-ol[1] and c) phenol.

Excited State 1: Singlet-A 2.0930 eV 592.38 nm f=0.0797

24_4-O4_CH3_HOMO

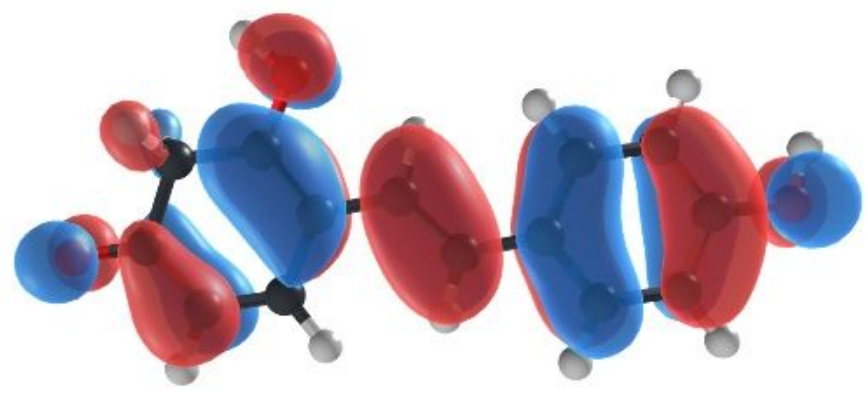

24_4-O4_CH3_LUMO

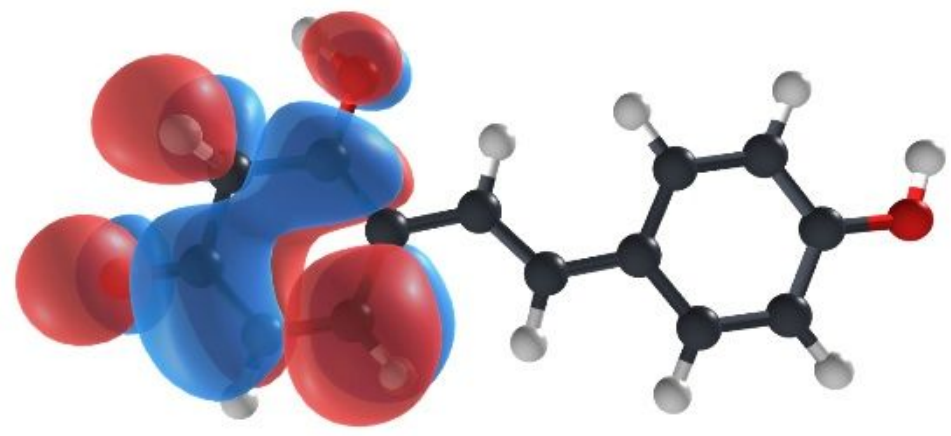

Excited State 1: Singlet-A 2.2909 eV 541.21 nm f=0.1389

24_4-O4-CH5_HOMO

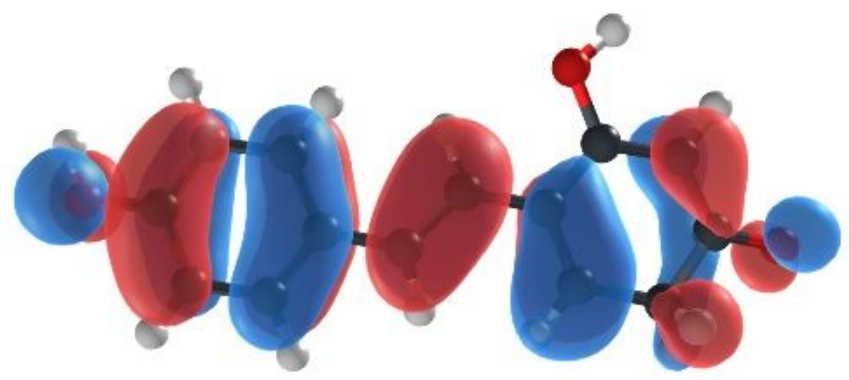

24_4-O4_CH5_LUMO

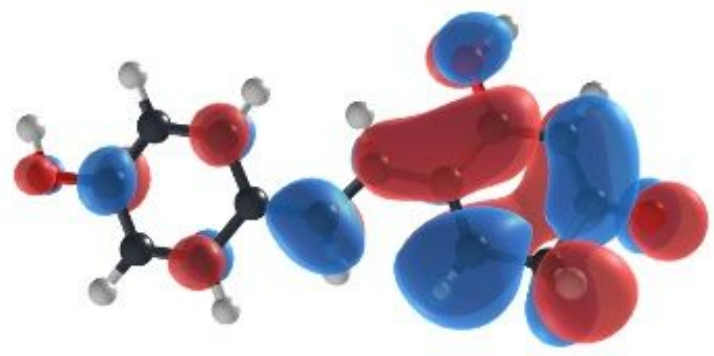

Excited State 1: Singlet-A 1.8513 eV 669.71 nm f=0.0468

24-O4-CH3_HOMO

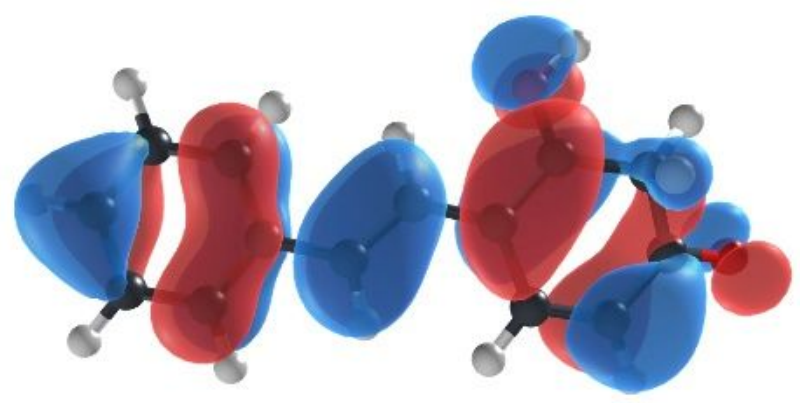

24-O4-CH3_LUMO

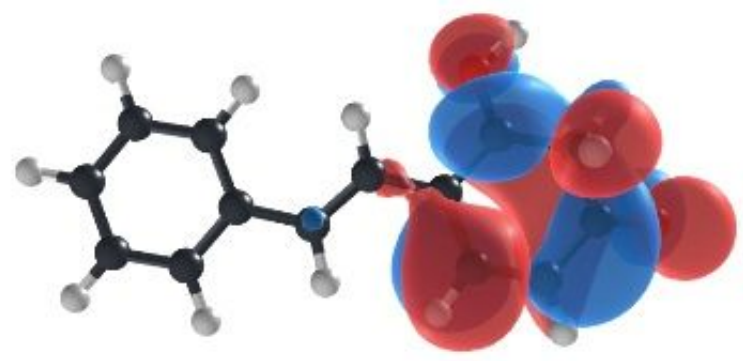

Excited State 1: Singlet-A 2.1445 eV 578.14 nm f=0.0779

24-O4-CH5_HOMO

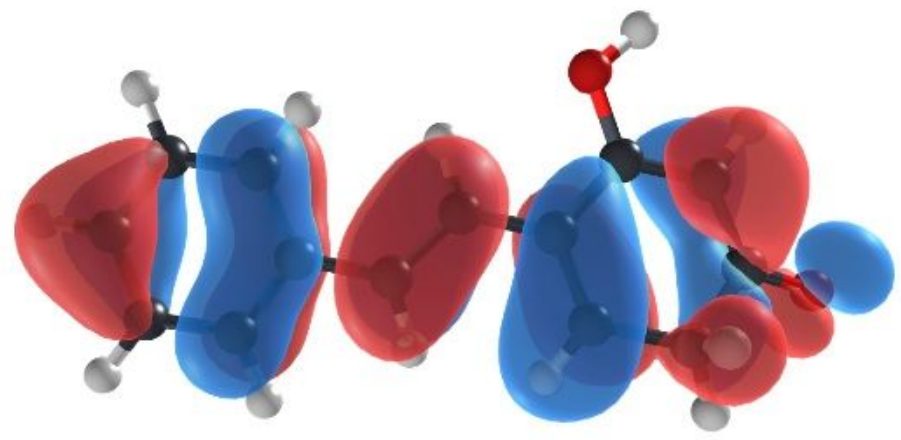

24-O4-CH5_LUMO

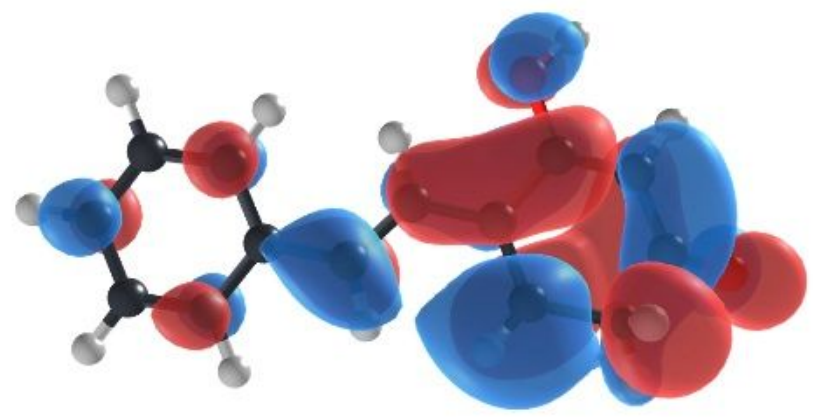

Excited State 1: Singlet-A 2.7226 eV 455.38 nm f=0.2493

2-O2-CH5_HOMO

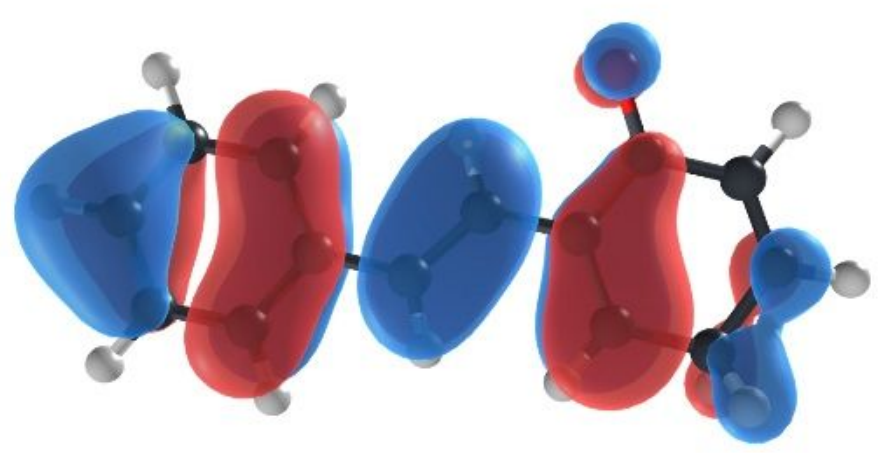

2-O2-CH5_LUMO

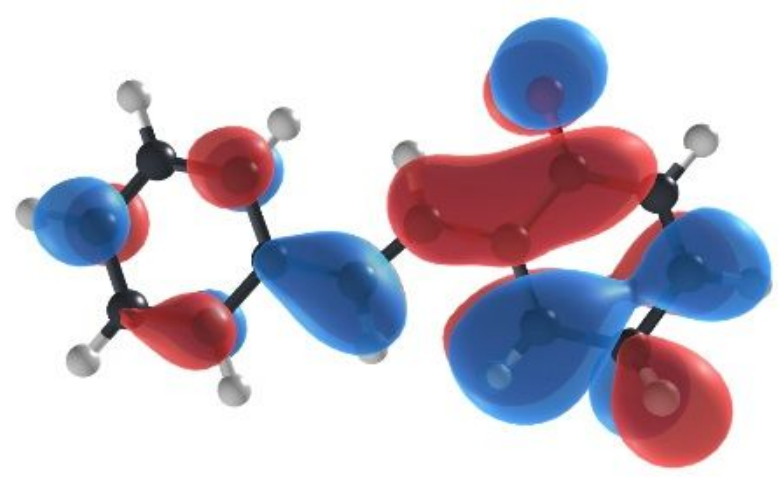

Figure S4. Frontier molecular orbitals with corresponding energy in electronvolt (eV), nanometers (nm), oscillator strength of first electron transition in first excited state.

Excited State 1: Singlet-A' 2.4555 eV 504.92 nm f=0.1768

35_2-O3_HOMO 35_2-O3_LUMO

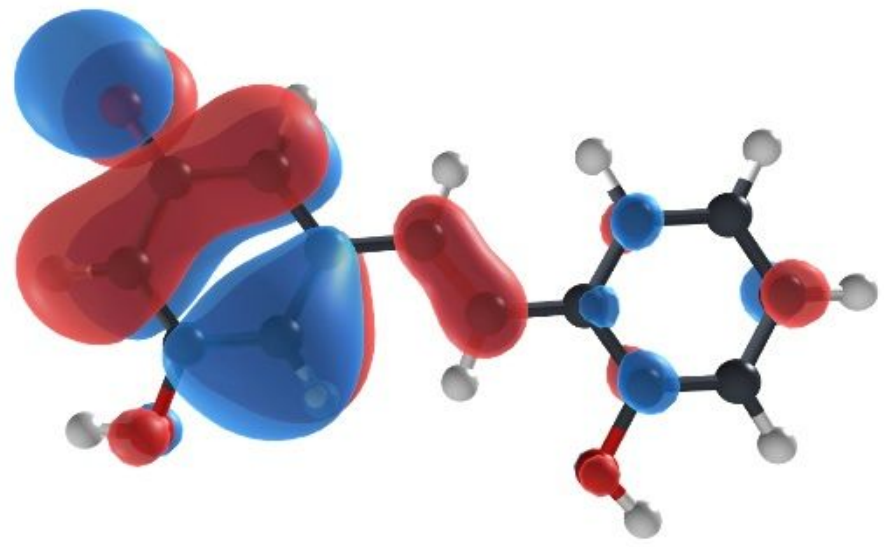
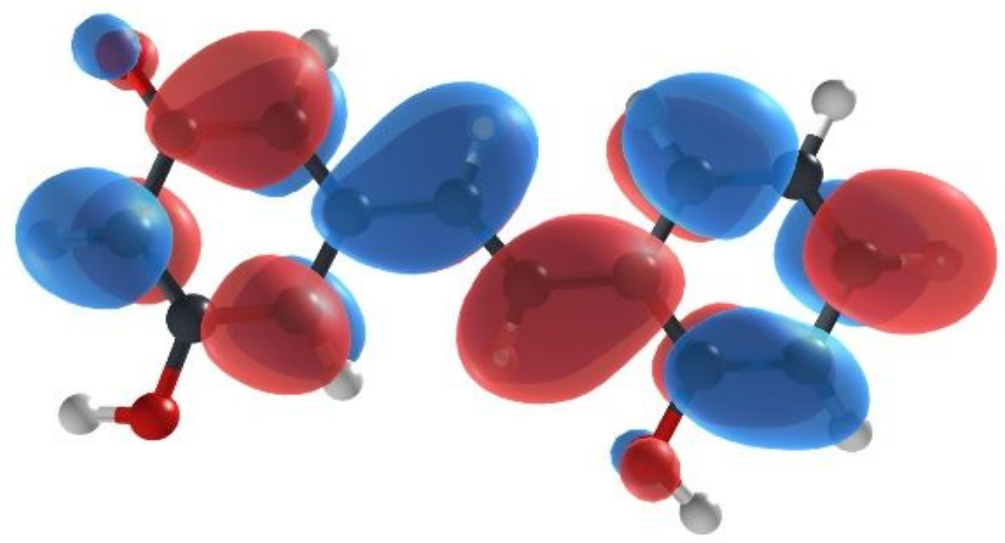

Excited State 1: Singlet-A' 2.5183 eV 492.33 nm f=0.1638

35_2-O5_HOMO 35_2-O5_LUMO

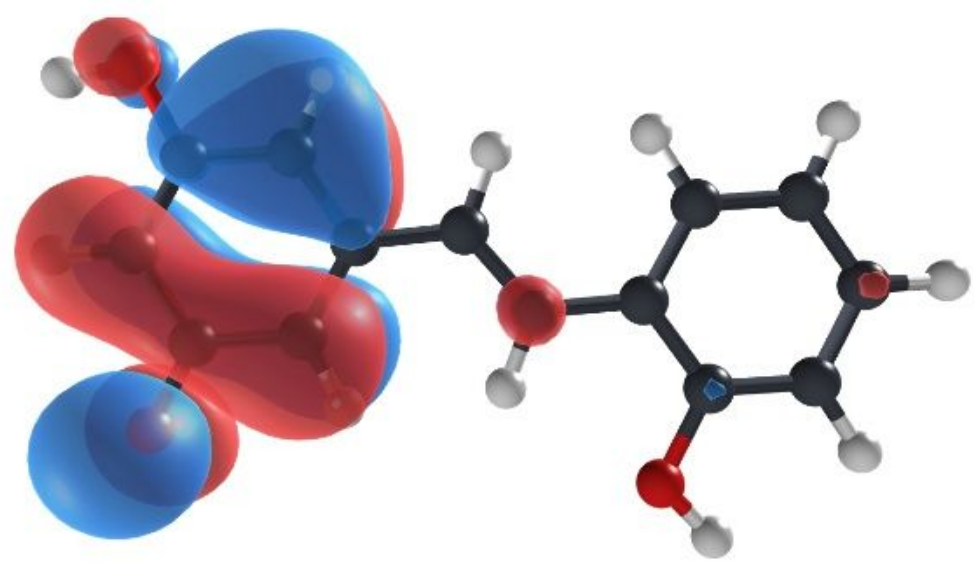
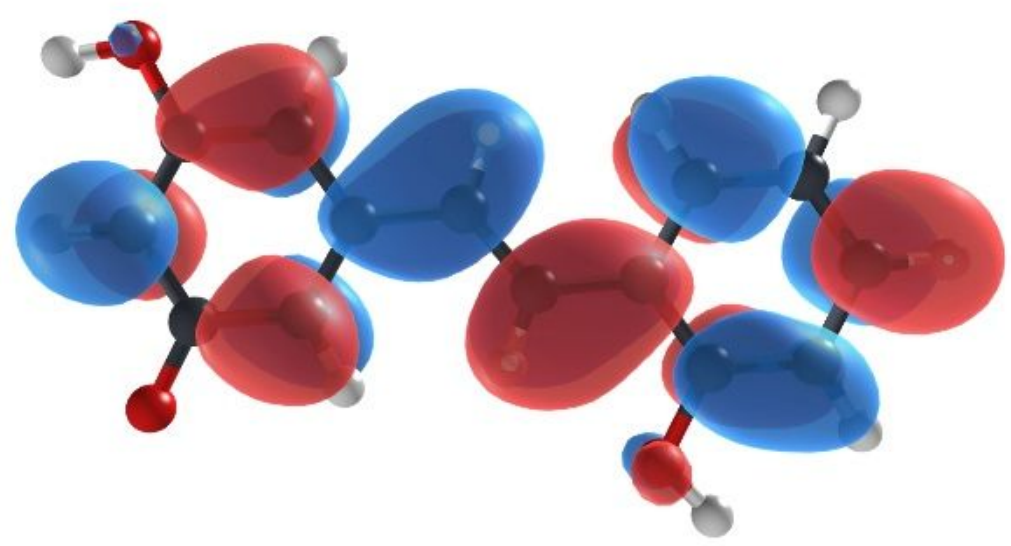

Excited State 1: Singlet-A' 2.4517 eV 505.71 nm f=0.1510

35_35-O3_HOMO 35_35-O3_LUMO

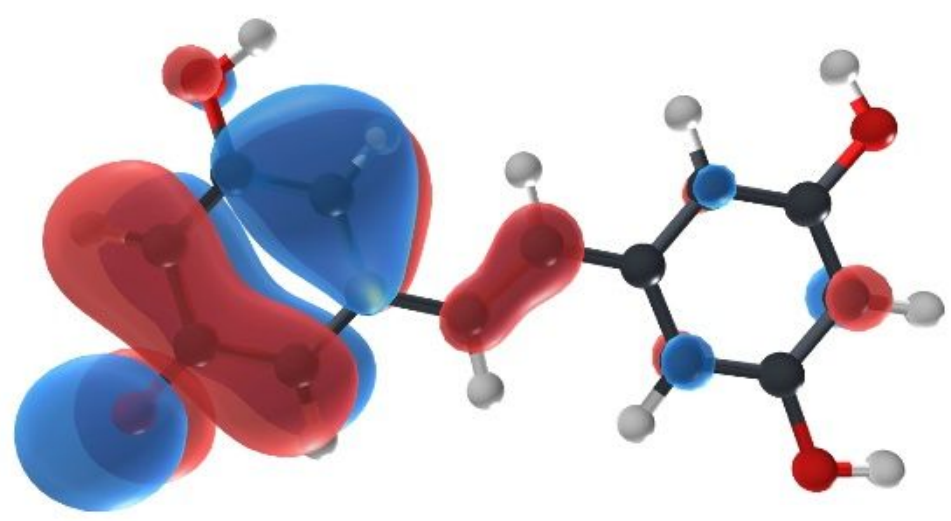
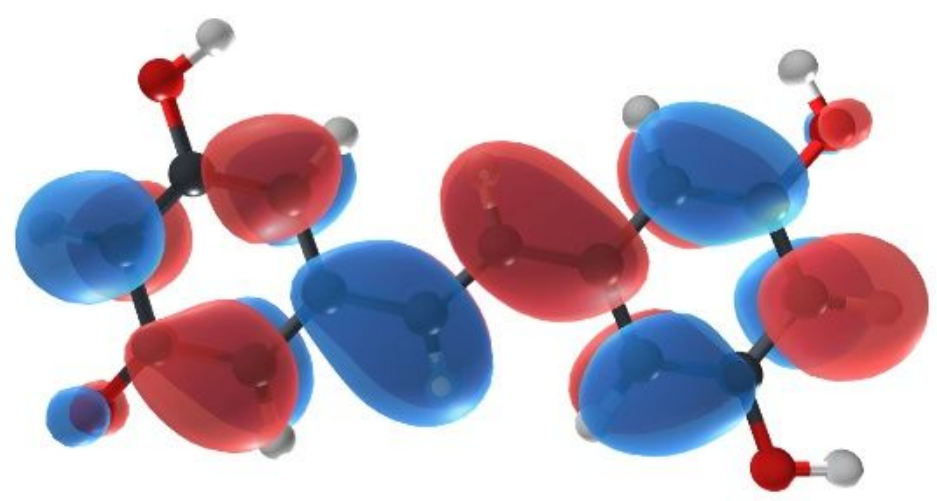

Excited State 1: Singlet-A' 2.4419 eV 507.73 nm f=0.1384

35_35-O3c_HOMO 35_35-O3c_LUMO

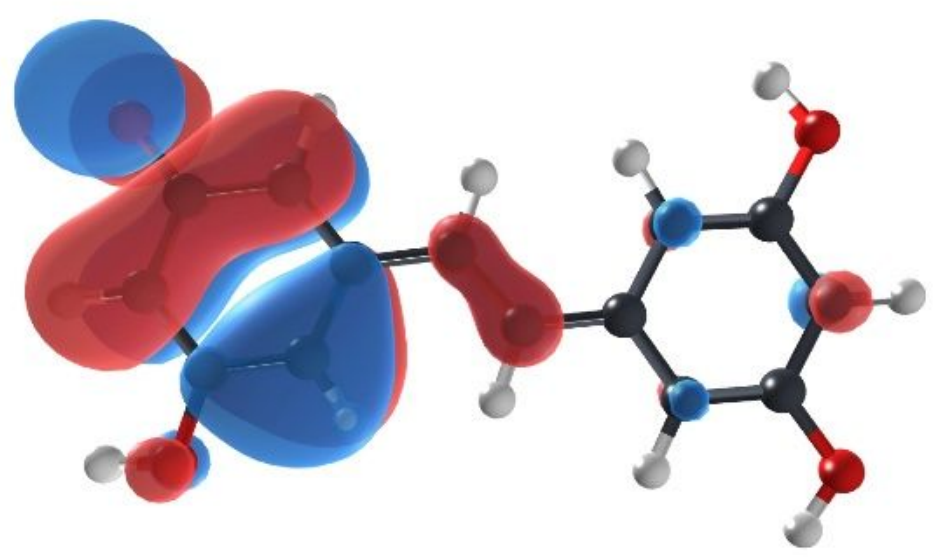
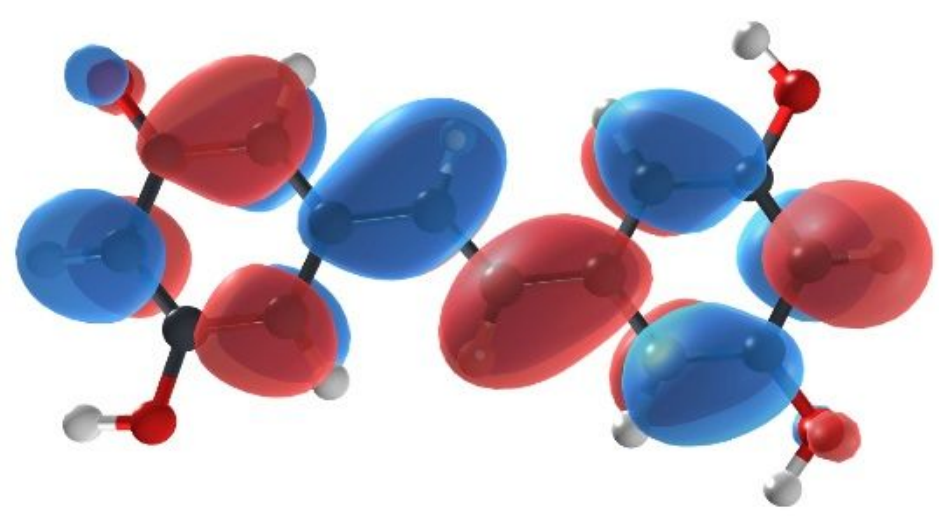

Figure S4 (continued). Frontier molecular orbitals with corresponding energy in electronvolt (eV), nanometers (nm), oscillator strength of first electron transition in first excited state.

Excited State 1: Singlet-A' 2.5066 eV 494.64 nm f=0.1507

35_35-O5_HOMO 35_35-O5_LUMO

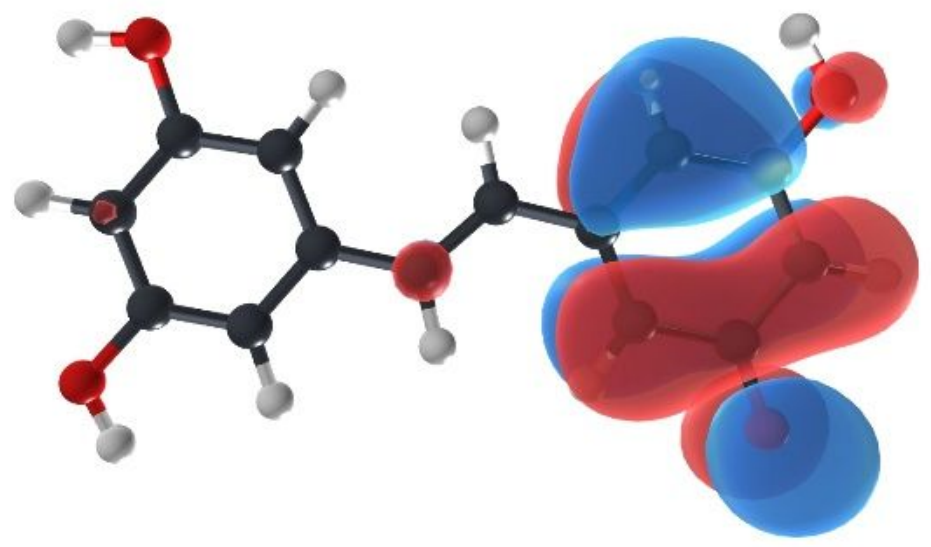

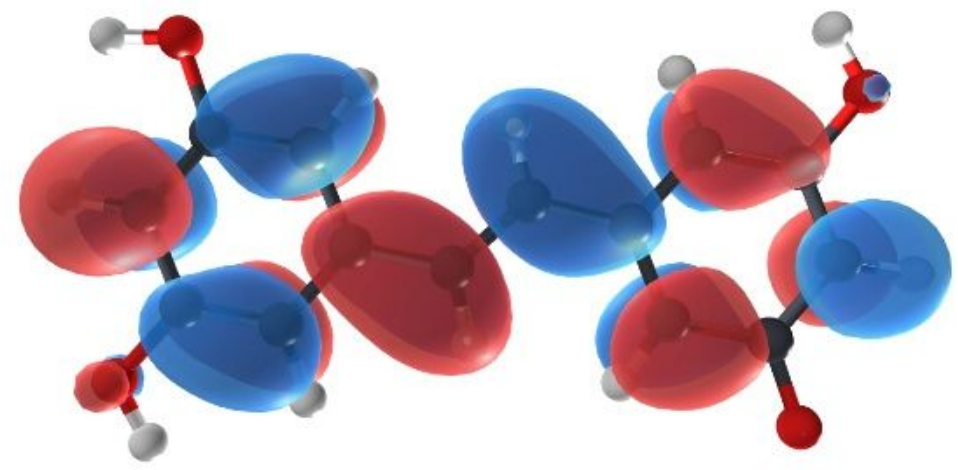

Excited State 1: Singlet-A' 2.5039 eV 495.16 nm f=0.1518

35_35-O5c_HOMO 35_35-O5c_LUMO

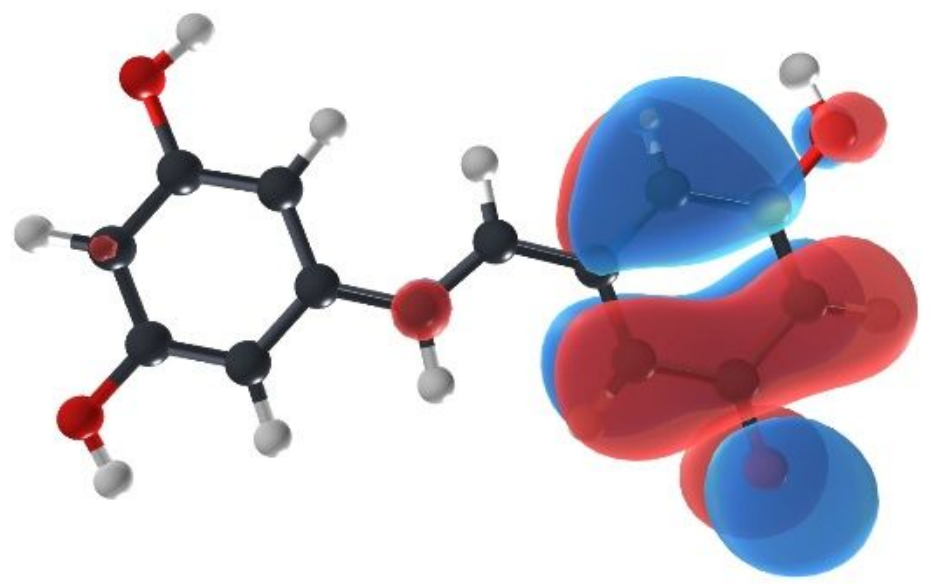

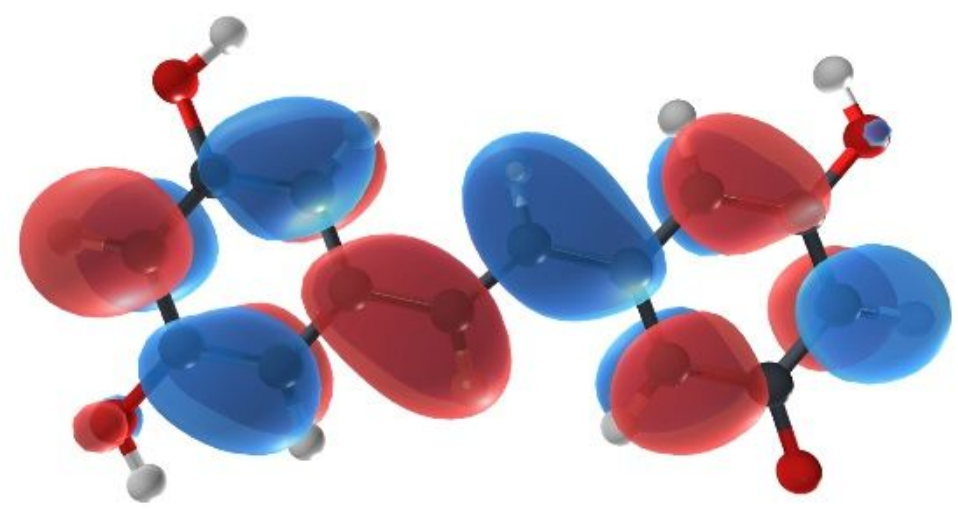

Excited State 1: Singlet-A 2.0878 eV 593.86 nm f=0.0311

35_4-O4c-CH3c_HOMO 35_4-O4c-CH3c_LUMO

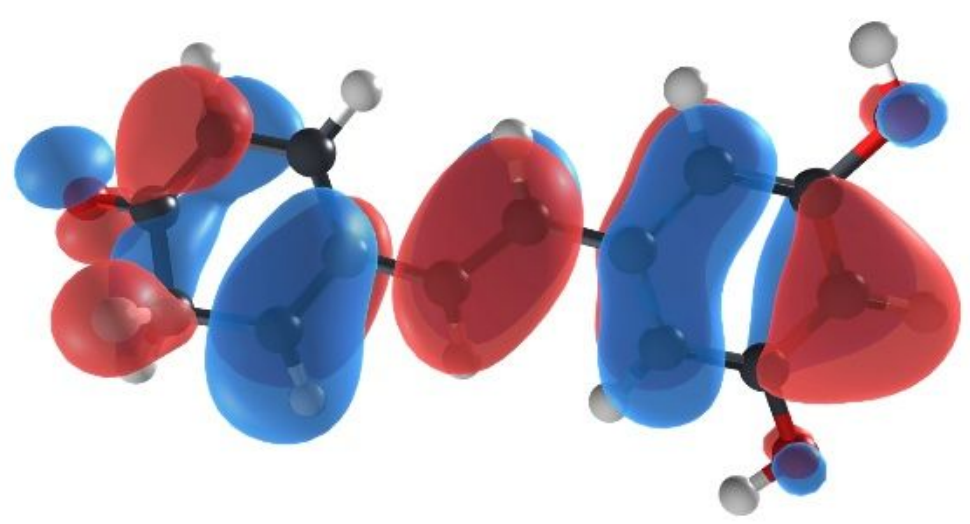

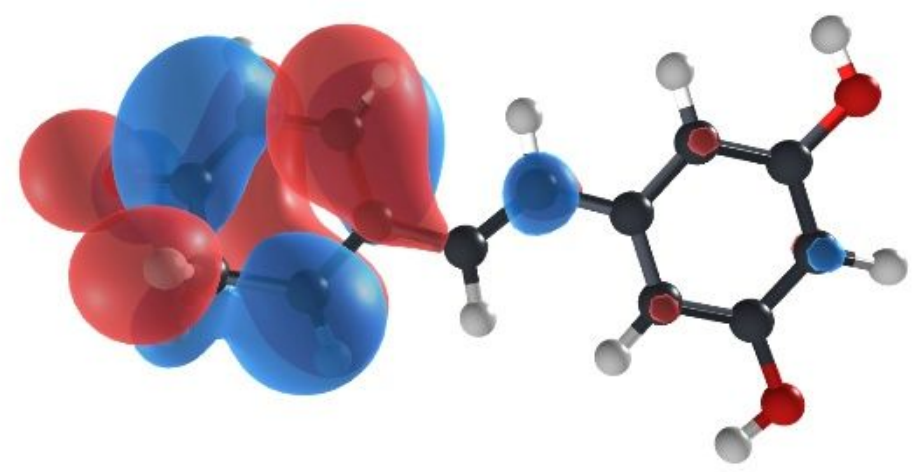

Excited State 1: Singlet-A 1.9823 eV 625.47 nm f=0.0401

35_4-O4c-CH5c_HOMO 35_4-O4c-CH5c_LUMO

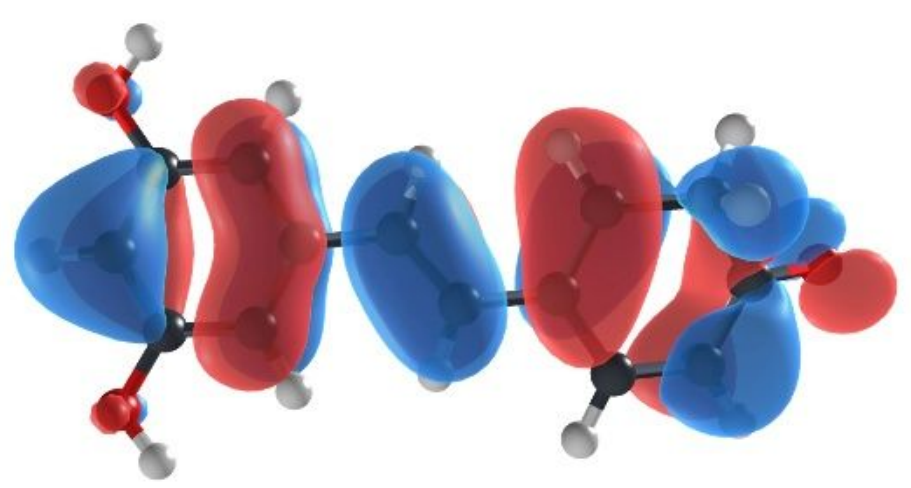

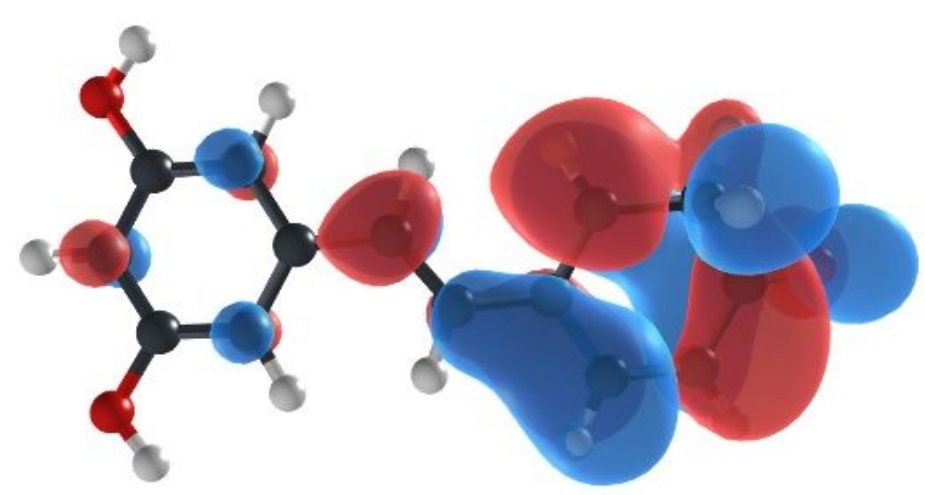

Figure S4 (continued). Frontier molecular orbitals with corresponding energy in electronvolt (eV), nanometers (nm), oscillator strength of first electron transition in first excited state.

Excited State 1: Singlet-A' 2.4522 eV 505.60 nm f=0.1411

35_5-O3_HOMO 35_5-O3_LUMO

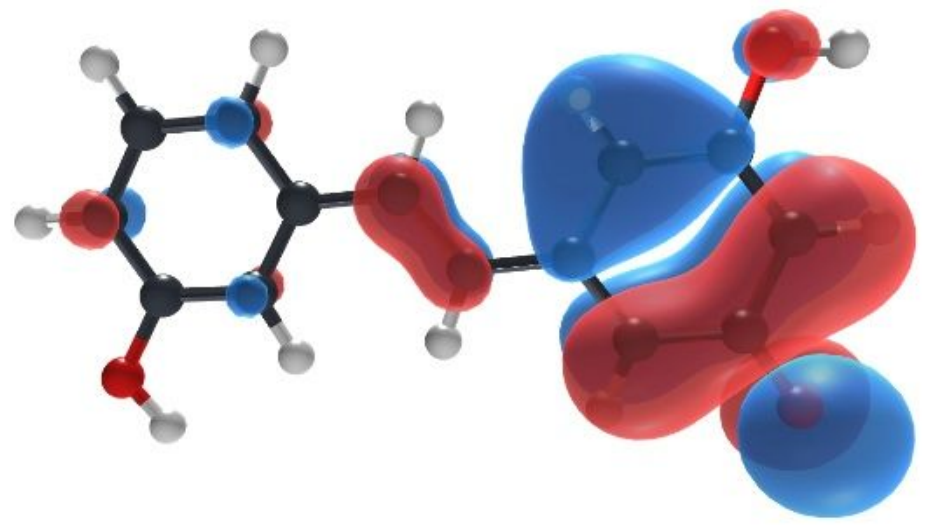

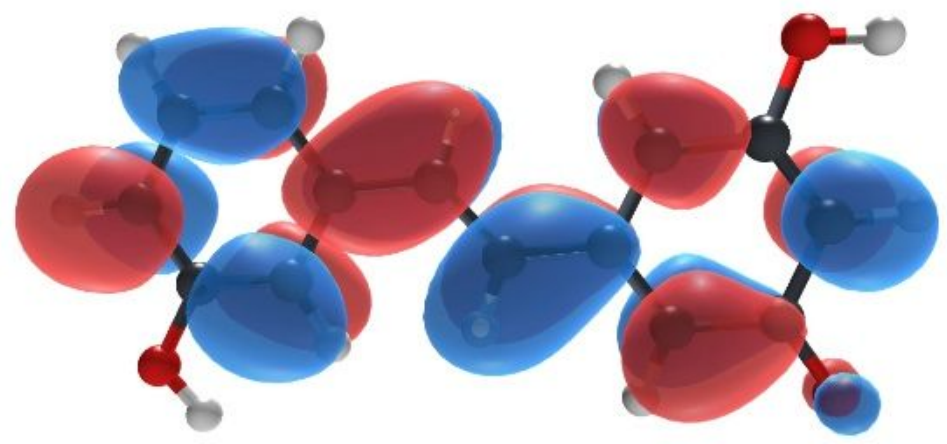

Excited State 1: Singlet-A' 2.5094 eV 494.08 nm f=0.1539

35_5-O5_HOMO 35_5-O5_LUMO

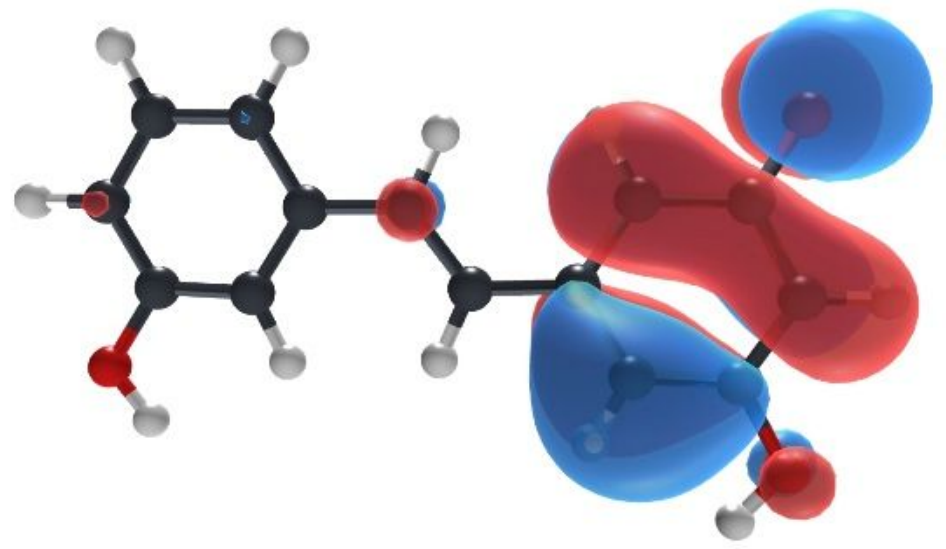

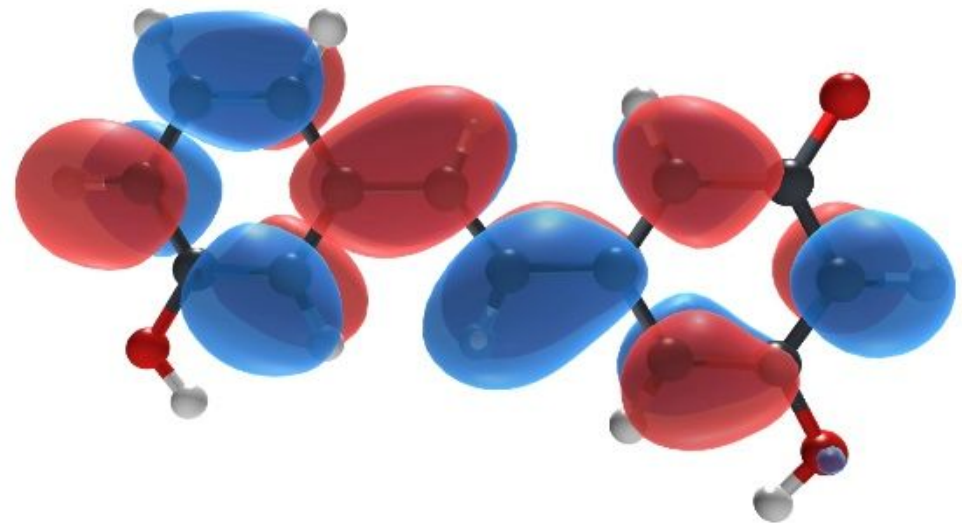

Excited State 1: Singlet-A 2.2273 eV 556.66 nm f=0.0413

35_6-O6c-CH3c_HOMO 35_6-O6c-CH3c_LUMO

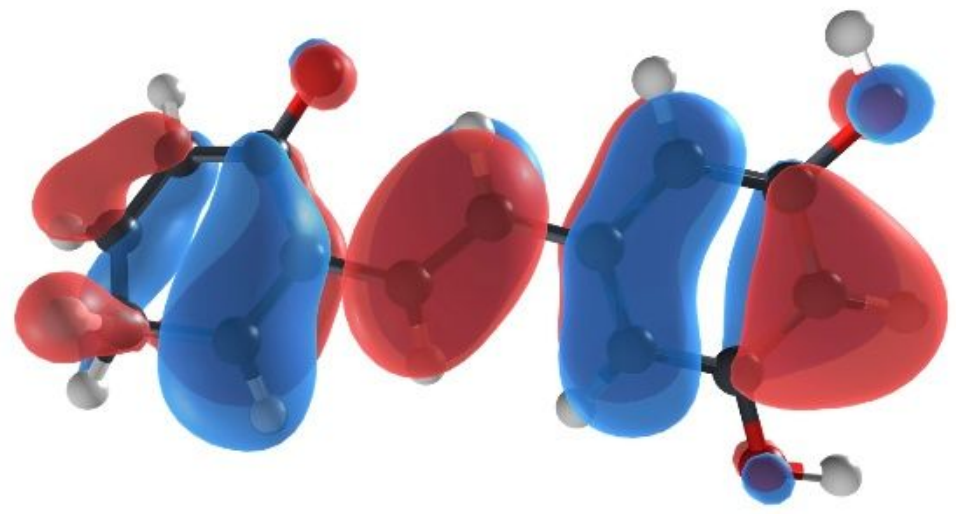

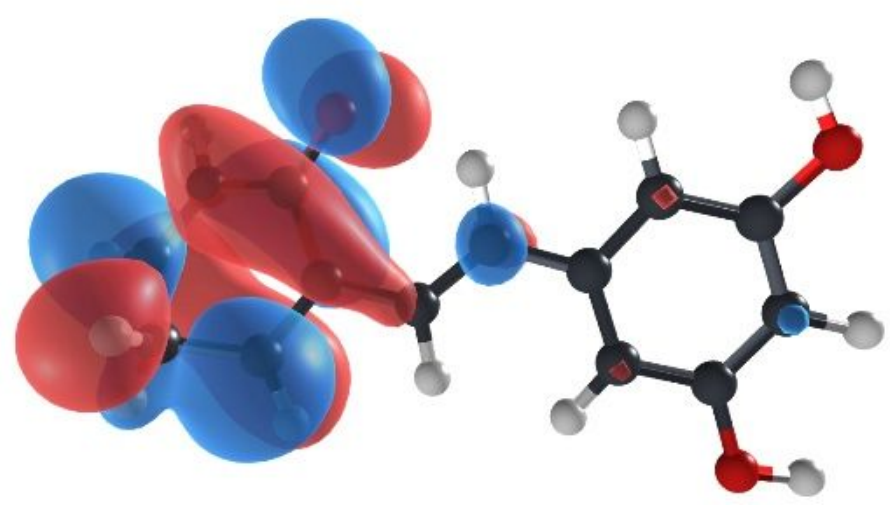

Excited State 1: Singlet-A 2.0754 eV 597.41 nm f=0.0310

4-O4-CH3_HOMO 4-O4-CH3_LUMO

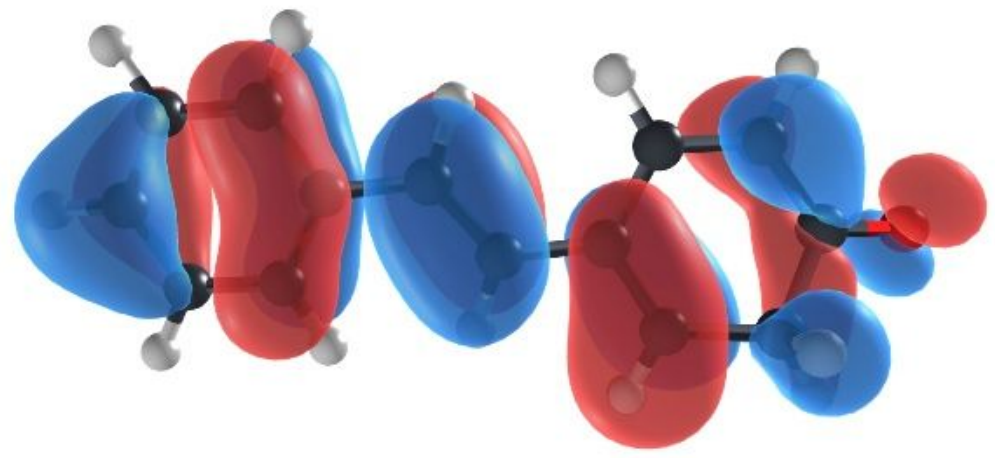

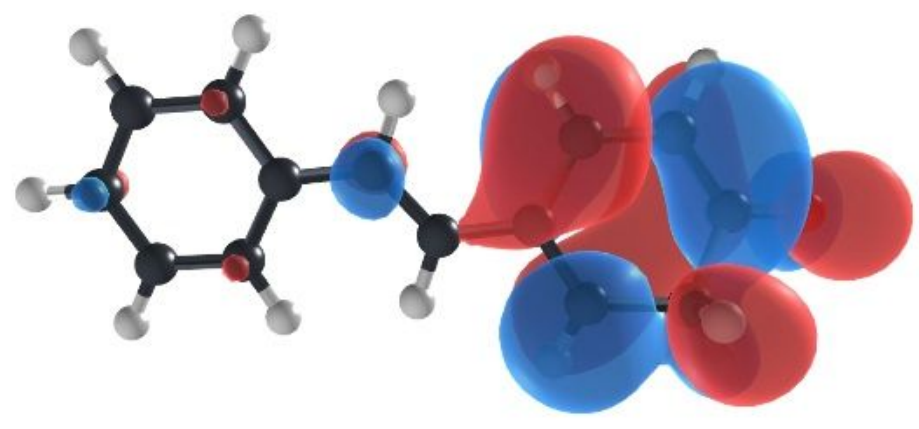

Excited State 1: Singlet-A 1.9672 eV 630.25 nm f=0.0390

4-O4-CH5_HOMO 4-O4-CH5_LUMO

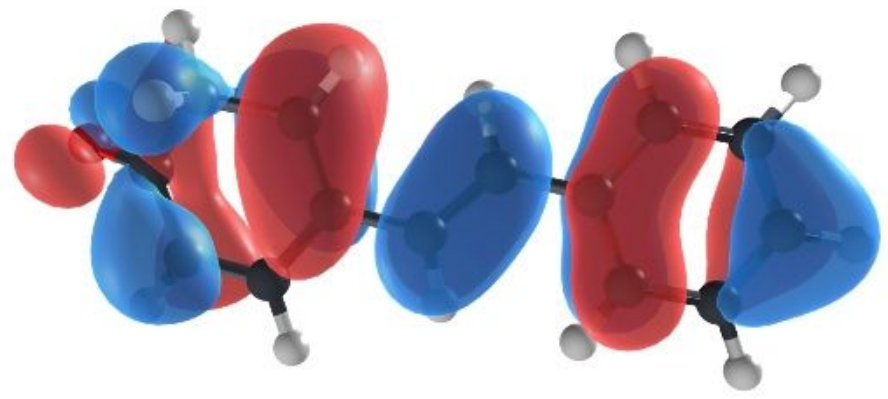

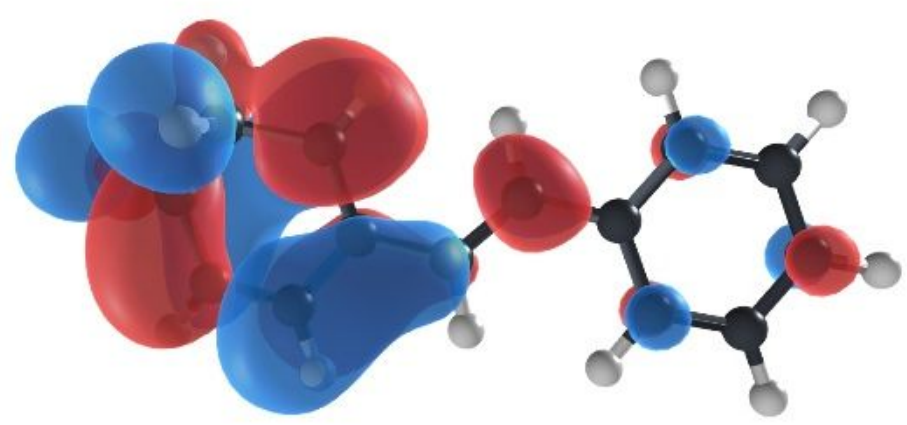

Excited State   1:   Singlet-A   2.1711 eV 571.06 nm f=0.0407

6-O6_CH3_HOMO   6-O6_CH3_LUMO

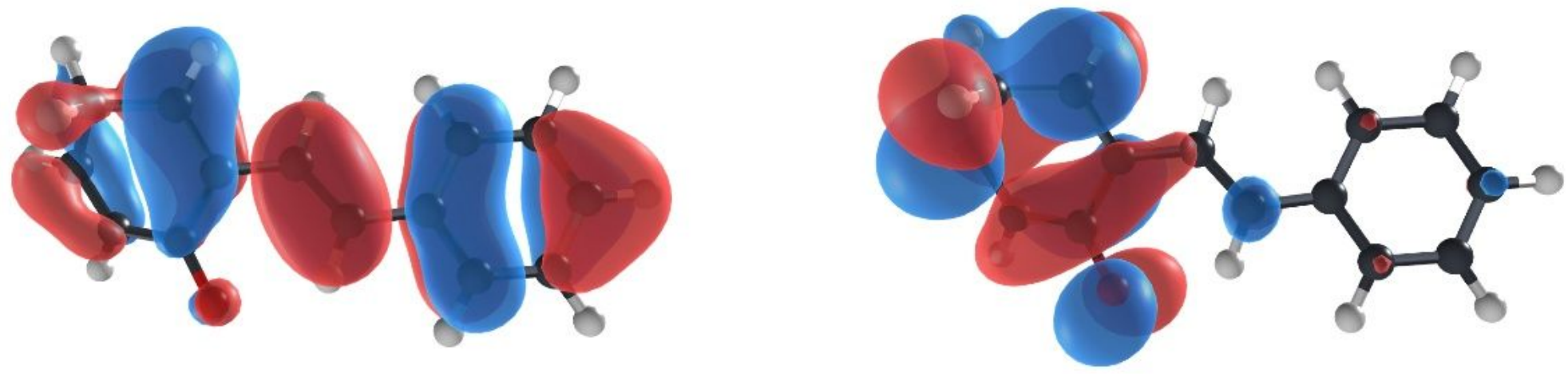

Figure S4. Frontier molecular orbitals with corresponding energy in electronvolt (eV), nanometers (nm), oscillator strength of first electron transition in first excited state.

To the best of our knowledge, only two experimental data points regarding the pK_a* values of stilbenols are currently available in the scientific literature, and these are reported to have low measurement uncertainty. Specifically, these are molecule **4** with a pK_a* value of 2.0 and **36_4** (resveratrol) with a value of 2.5. If we were to consider these two values, linear function for the evaluation of scaled p$K_{a,sc}$* values

$$pK_{a,sc}^* = 0.515 \times pK_a^* - 2.330 \quad \text{(S1)}$$

The evaluated scaled values are presented in **Figure S5**.

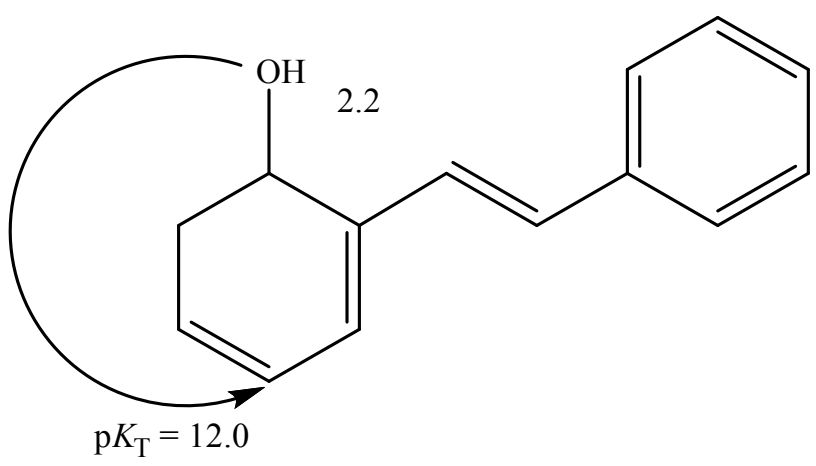

2

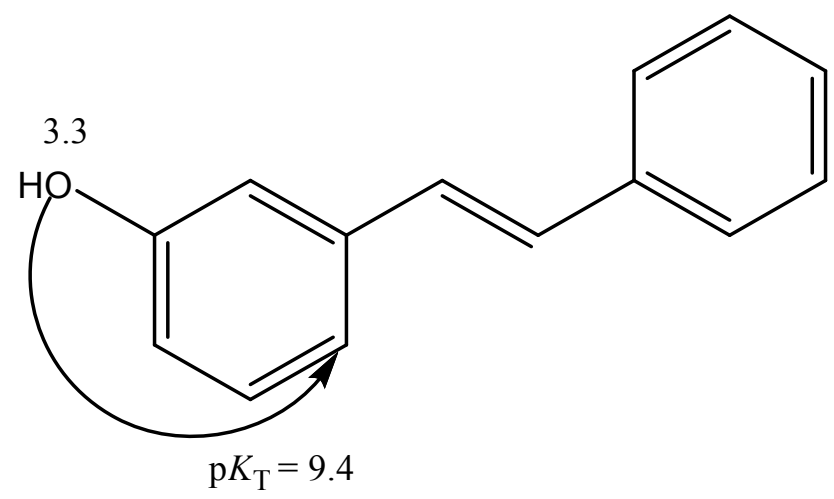

3

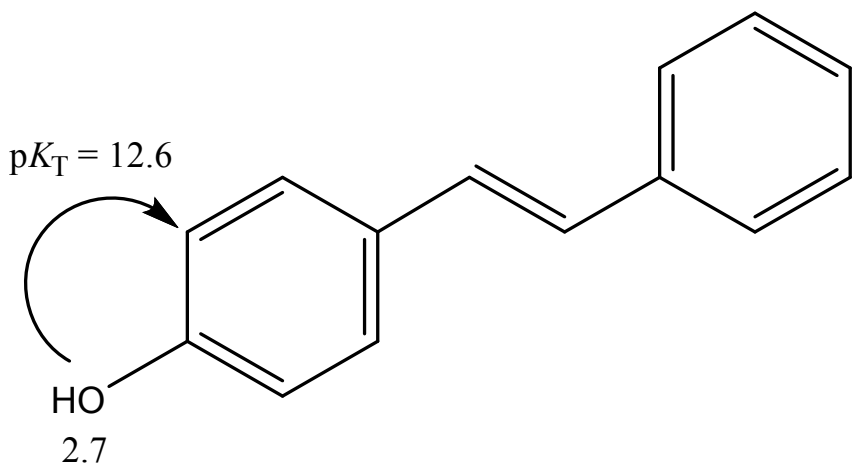

4

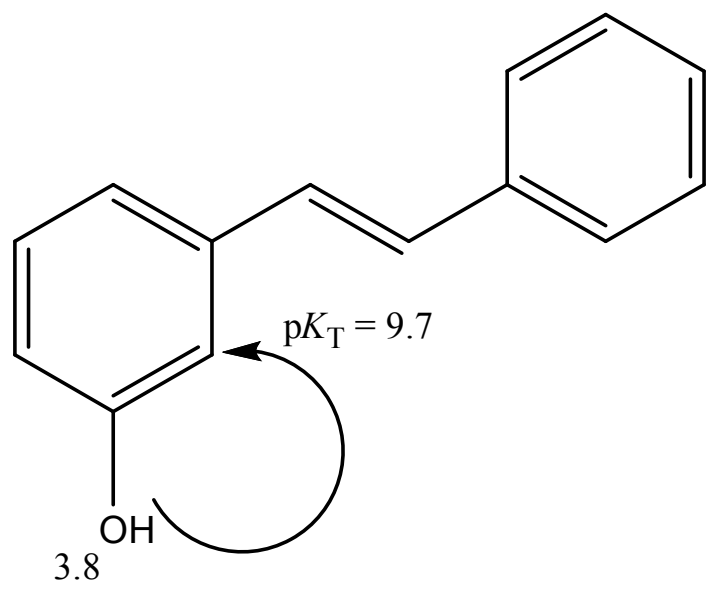

5

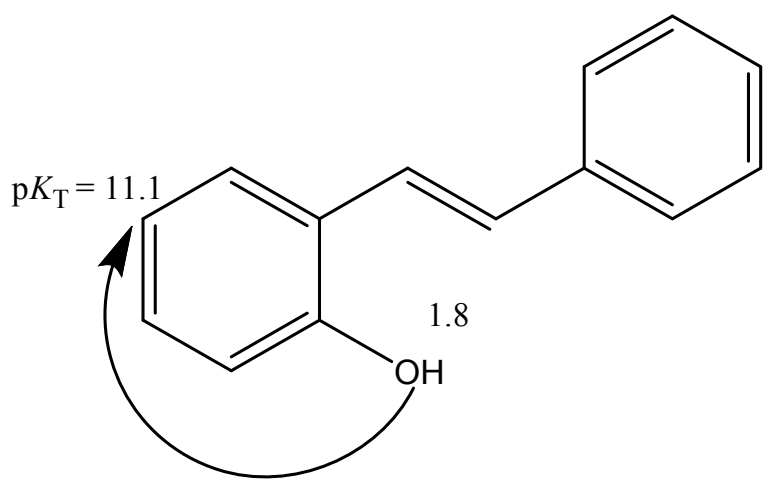

6

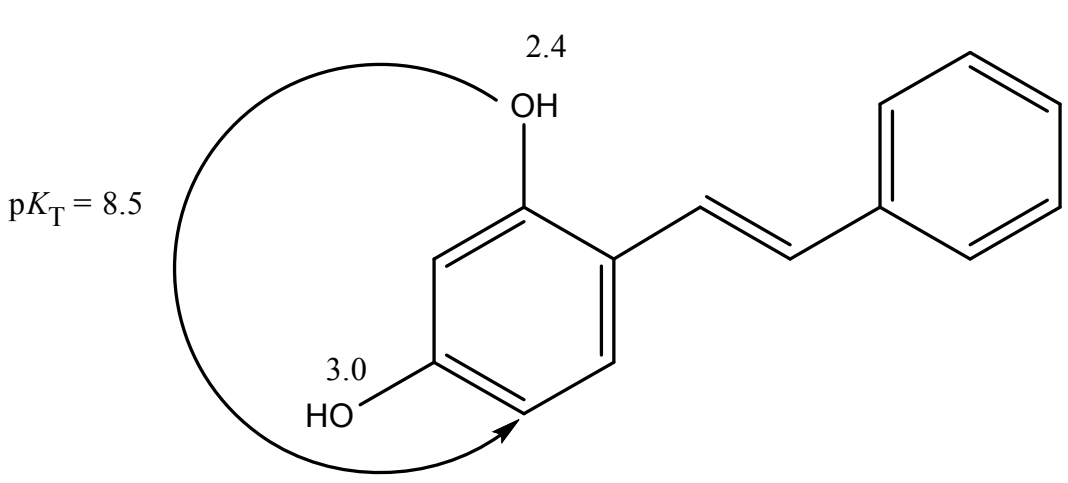

24

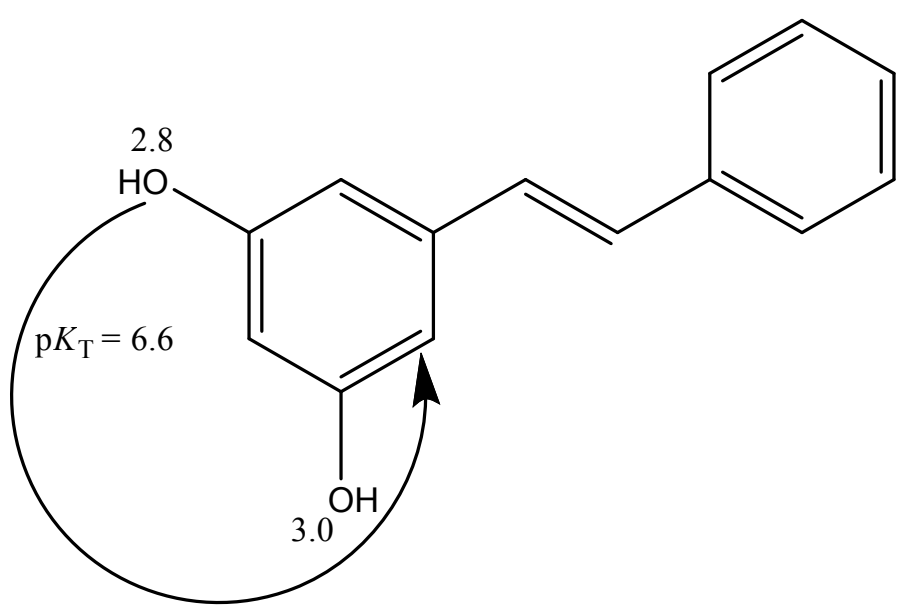

35

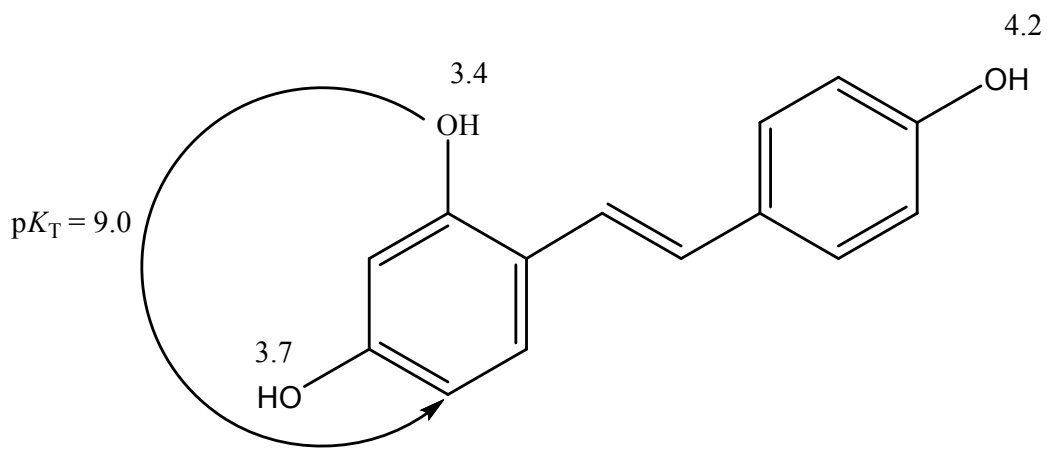

24_4

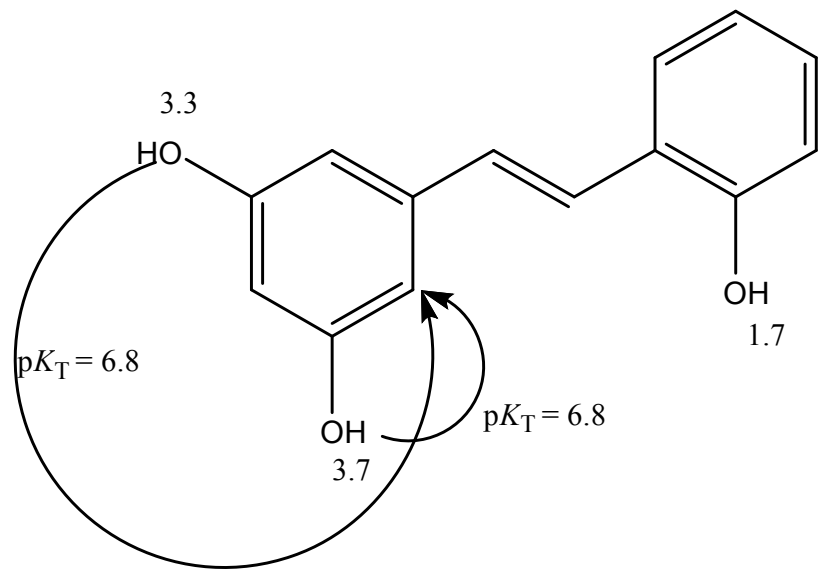

35_2

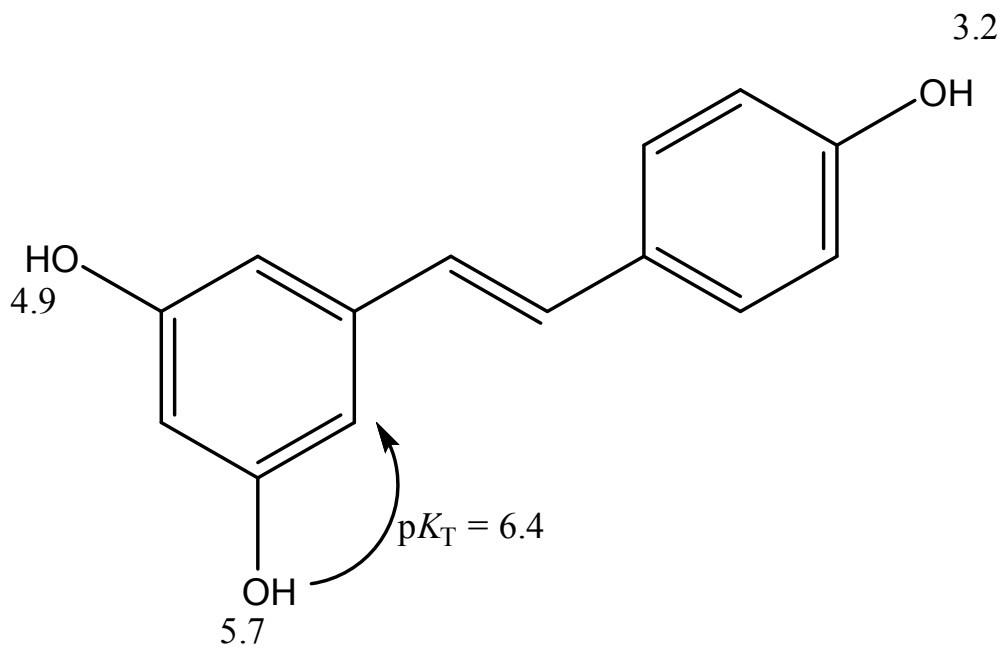

35_4

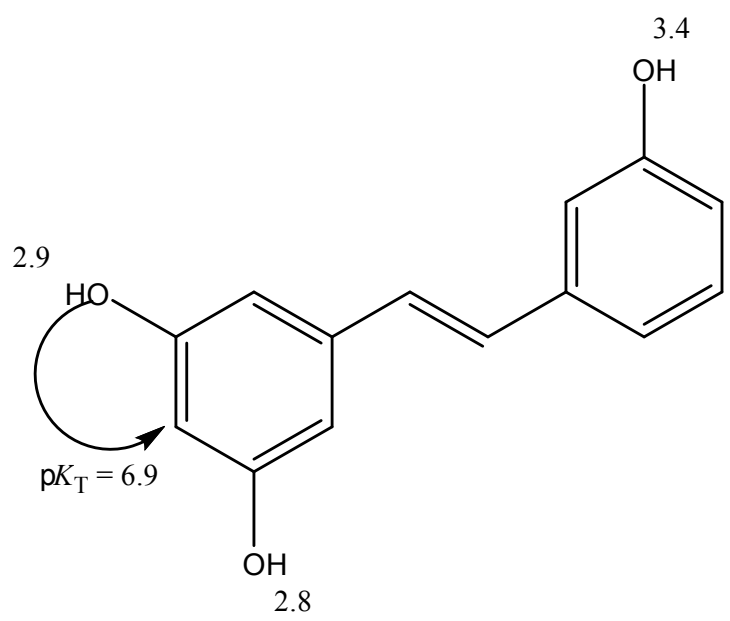

35_5

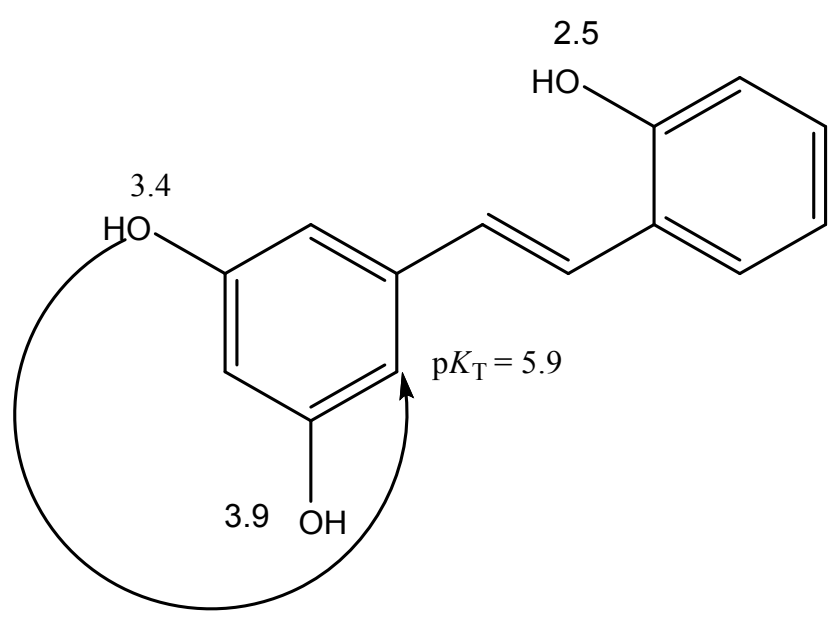

35_6

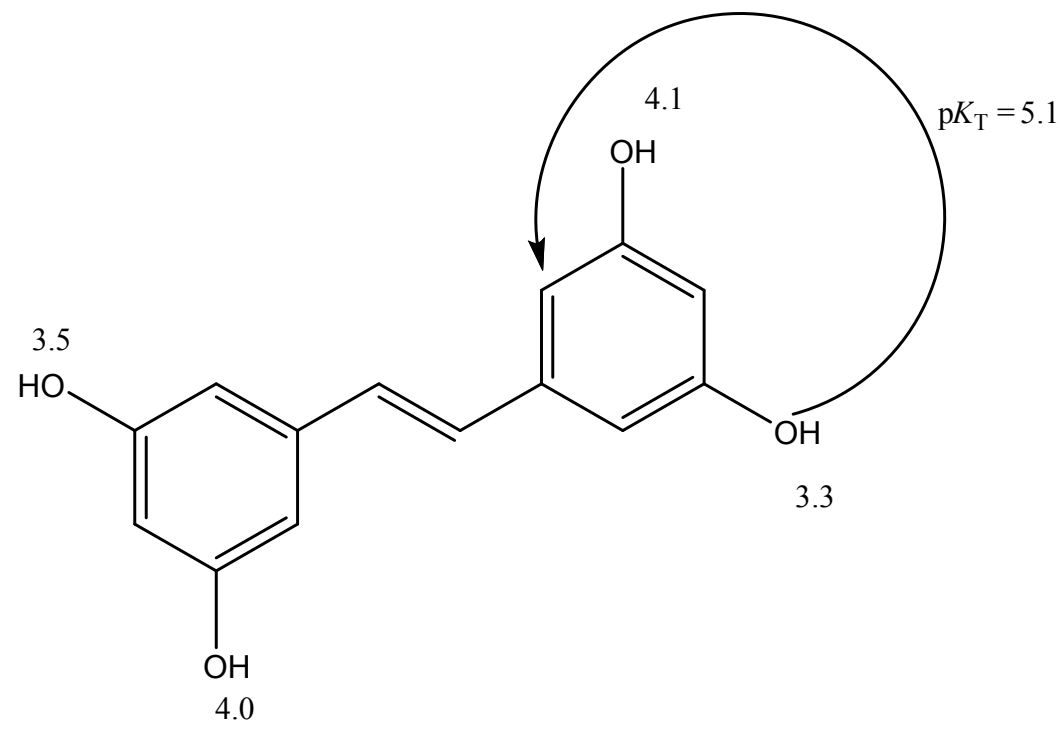

35_35

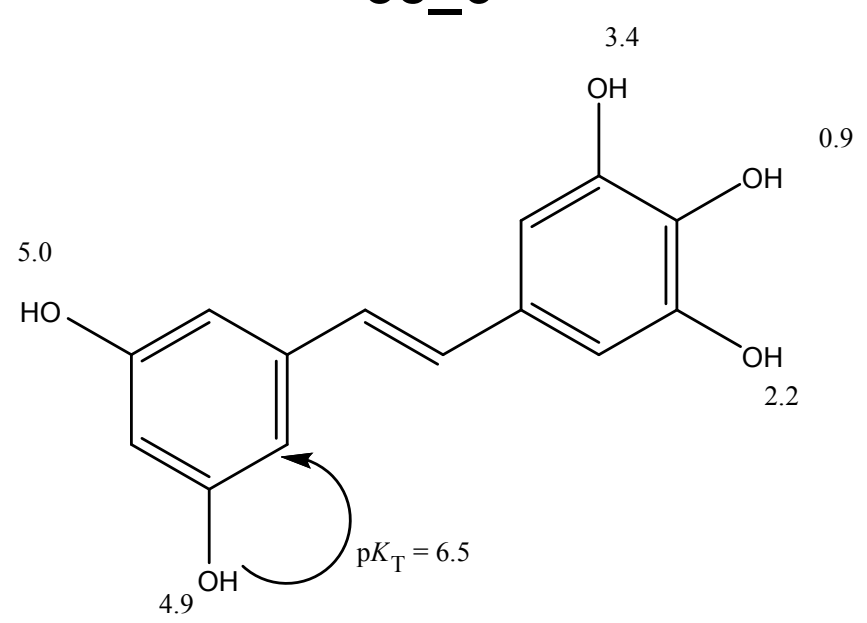

35_345

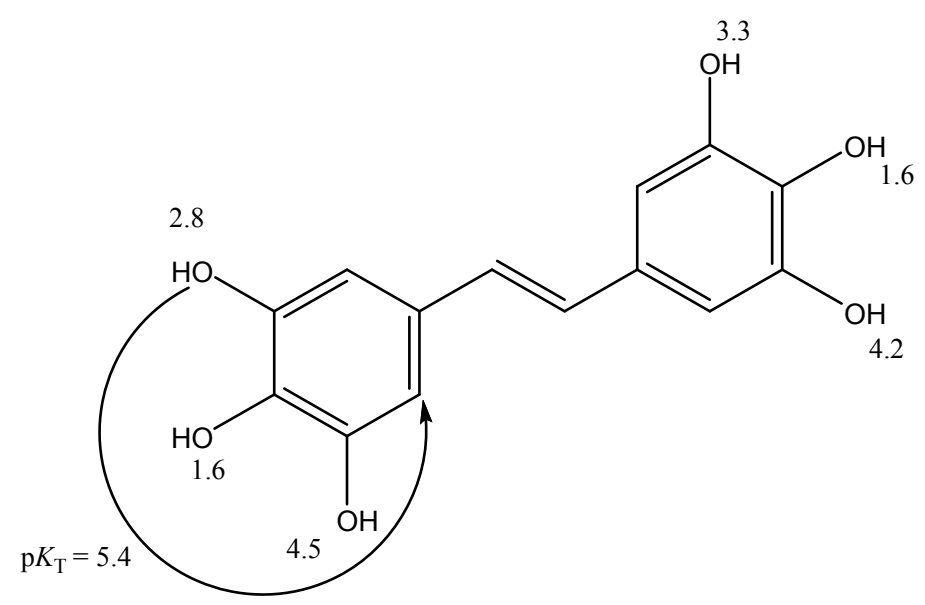

345_345

Figure S5. The estimated $pK^*_{a,sc}$ values according to the Equation S1 and the scaled $pK_{T,sc}$ values for energetically preferred keto-enol tautomerisation equilibria.

Table S1. Calculated reaction Gibbs free energies (see Eq. 2), theoretical pK_a and experimental p$K_{a,exp}$ values.

molecule	$\Delta_r G$ / kJ mol^{-1}	pK_a	p$K_{a,exp}$
**1-naphthol**	1206	16.3	9.4[2]
**2-naphthol**	1209	16.9	9.6[2]
**4**	1205	16.1	9.3[3]
**Phenol**	1212	17.3	9.8[2]

Table S2. Calculated reaction Gibbs free energies, theoretical pK_T and experimental p$K_{T,exp}$ values.

**molecule/reaction**	**ΔG / kJ mol^{-1}**		**pK_T**		**p$K_{T,exp}$**	
	ortho	***para***	***ortho***	***para***	***ortho***	***para***
1-naphthol	31	28	5.4	4.9	7.1[1]	6.2[1]
Phenol	64	59	11.3	10.3	12.7[4]	11.0[4]

Table S3. The scaled p$K_{T,sc}$ constants for the prototropic equilibrium between hydroxy group and C-protonated atoms for mono- and bisubstituted molecules.

	**2**	**3**	**4**	**5**	**6**	**24**	**35**
**C2=O**							
**C2´H_2**	23.6					21.0	
**C3H_2**	12.4					9.7	
**C4´H_2**	21.4					18.7	
**C5H_2**	12.0					8.5	
**C6´H_2**	23.0					20.2	
**C3=O**							
**C2H_2**		10.9					8.0
**C4H_2**		10.4					8.9
**C6H_2**		9.4					6.6
**C4=O**							
**C2´H_2**			22.1				
**C3H_2**			12.6			9.9	
**C4´H_2**			19.8				
**C5H_2**			13.3			9.8	
**C6´H_2**			21.4				
**C5=O**							
**C2H_2**				10.4			8.3
**C4H_2**				11.4			9.3
**C6H_2**				9.7			6.9
**C6=O**							
**C2´H_2**					22.9		
**C3H_2**					11.1		
**C4´H_2**					20.7		
**C5H_2**					12.1		
**C6´H_2**					22.4		

Table S4. The scaled p$K_{T,sc}$ constants for the prototropic equilibrium between hydroxy group and C-protonated atoms for more substituted molecules.

	24_4	35_2	35_4	35_5	35_6	35_35	35_345	345_345
C2=O								
C2´H2	22.3							
C3H2	9.4							
C4´H2								
C5H2	9.0							
C6´H2	21.4							
C3=O								
C2H2		8.3	7.8	6.9	7.7	7.1	8.3	6.9
C4H2		8.9	8.1	7.2	8.3	7.1		
C6H2		6.8	7.1		5.9	5.9	6.9	5.4
C4=O								
C2´H2								15.0
C3H2	9.9							
C5H2	9.9							
C6´H2	20.7							14.4
C5=O								
C2H2		7.6	8.2	7.3	8.0	7.1	8.4	8.6
C4H2		9.3	9.0	7.9	8.9	8.0		
C6H2		6.8	6.4		6.5	5.6	6.5	6.9
C2´=O								
C2H2		16.6						
C3´H2								
C4H2		16.2						
C5´H2								
C6H2		15.8						
C3´=O								
C2´H2						6.8	7.8	8.8
C4´H2						7.3		
C6´H2						5.1	7.2	7.2
C4´=O								
C2H2			16.6				14.4	14.8
C3´H2			13.0					
C4H2							12.7	
C5´H2			14.1					
C6H2	22.3		15.6				13.6	14.7
C5´=O								
C2´H2				9.7		7.1	9.4	6.7
C4´H2				10.8		7.9		
C6´H2				9.2		5.3	7.4	5.5
C6´=O								
C2H2					16.2			
C3´H2					10.1			
C4H2					15.2			
C5´H2					10.6			
C6H2					14.9			

References

(1) Gut, I. G.; Scheibler, L. C.; Wirz, J. Flash Photolytic Generation of Two Keto Tautomers of 1-Naphthol in Aqueous Solution: Kinetics and Equilibria of Enolization. *Photochem. Photobiol. Sci.* **2010**, *9* (7), 901–907.

(2) Chemistry, T. *The Chemistry of Phenols Patai Series : The Chemistry of Functional Groups*; 2003.

(3) Lewis, F. D.; Crompton, E. M. Hydroxystilbene Isomer-Specific Photoisomerization versus Proton Transfer. **2003**, 4044–4045.

(4) Capponi, M.; Gut, I. G.; Hellrung, B.; Persy, G.; Wirz, J. Ketonization Equilibria of Phenol in Aqueous Solution. *Can. J. Chem.* **1999**, *77* (5–6), 605–613.
